# Multi-modal effects of 1B3, a novel synthetic miR-193a-3p mimic, support strong potential for therapeutic intervention in oncology

**DOI:** 10.18632/oncotarget.27894

**Published:** 2021-03-02

**Authors:** Bryony J. Telford, Sanaz Yahyanejad, Thijs de Gunst, Harm C. den Boer, Rogier M. Vos, Marieke Stegink, Marion T.J. van den Bosch, Mir Farshid Alemdehy, Laurens A.H. van Pinxteren, Roel Q.J. Schaapveld, Michel Janicot

**Affiliations:** ^1^InteRNA Technologies BV, Utrecht, The Netherlands

**Keywords:** miR-193a-3p, microRNA mimic, microRNA delivery *in vivo*, pleiotropic mechanism of microRNA

## Abstract

Compelling evidence demonstrates that miR-193a-3p is a tumor suppressor microRNA in many cancer types, and its reduced expression is linked to cancer initiation and progression, metastasis, and therapy resistance. However, its mechanism of action is not consistently described between studies, and often contradicts the pleiotropic role of a microRNA in manipulating several different mRNA targets. We therefore comprehensively investigated miRNA-193a-3p's mode of action in a panel of human cancer cell lines, with a variety of genetic backgrounds, using 1B3, a synthetic microRNA mimic. Interestingly, the exact mechanism through which 1B3 reduced cell proliferation varied between cell lines. 1B3 efficiently reduced target gene expression, leading to reduced cell proliferation/survival, cell cycle arrest, induction of apoptosis, increased cell senescence, DNA damage, and inhibition of migration. SiRNA silencing of 1B3 target mRNAs further highlighted the advantage of the pleiotropic mechanism of 1B3 action, as repression of individual targets did not achieve the same robust effect on cell proliferation in all cell lines. Importantly, a novel lipid nanoparticle-based formulation of 1B3, INT-1B3, demonstrated marked anti-tumor activity as a single agent following systemic administration in tumor-bearing mice. Together, these data strongly support the development of 1B3 as a novel therapeutic agent for treatment of human cancer.

## INTRODUCTION

Despite decades of research and an unprecedented advancement of therapeutic options [1], cancer accounts for one in every six deaths worldwide, with ~18,000,000 new cases diagnosed in 2018 and expected to grow to ~30,000,000 by 2040^1^. Therefore, new therapeutic modalities are urgently required, particularly for tumors with high genetic heterogeneity and poor response to current treatments. Cancer is caused by the accumulation of dozens of mutations which lead to uncontrolled cell proliferation, but the precise biochemical changes and genetic background is unique to each individual cancer [2]. Even within a single patient, there is a high level of heterogeneity within tumors [3]. Cancer cells rely on a globally altered biochemical system to sustain proliferation, resist cell death, and to escape detection and clearance by the body’s immune system [2].

MicroRNAs (miRNAs) are naturally occurring short (~22 nucleotide) double stranded RNA molecules with a unique nucleotide sequence (seed sequence) that binds, at least partially, to complementary mRNA sequences. This leads to mRNA degradation or suppressed translation which then silences gene expression [4, 5]. The human genome encodes more than two thousand miRNAs which fine-tune normal cellular processes such as development and homeostasis [6]. Each miRNA recognizes and decreases (but does not completely extinguish) the expression of hundreds of target mRNAs. Fittingly, miRNAs are commonly dysregulated in cancer, and can act as either tumor suppressor or oncogenic miRNA [7, 8]. Antisense oligonucleotides against oncogenic miRNAs (antagomiRs to inhibit miRNA function) or synthetic copies of tumor suppressor miRNAs (miRNA mimics to increase miRNA levels) provide exciting novel approaches to cancer treatment [9, 10]. Because miRNAs simultaneously repress hundreds of target genes, they cause a cell-wide shift in biological network function. As cancer is a multifactorial disease, this pleiotropic effect of miRNAs may be more effective at targeting different aspects of cancer cell biology [11, 12]. In fact, as clinicians look more towards drug combinations for cancer treatment, antagomiRs and miRNA mimics represent obvious drug candidates for an all-in-one combination therapy. Despite this promise, miRNA therapeutics face challenges, particularly in ensuring the molecule is stable in the bloodstream and effectively delivered to the tumor tissue [12]. Currently, no miRNA mimic therapy has been approved by the FDA, although a few candidates are in preclinical and clinical development [13].

The therapeutic potential of miR-193a was identified in a high-throughput functional lentiviral-based screen of miRNAs in tumor cells and the 3p arm (miR-193a-3p) was subsequently demonstrated as the relevant active entity in screening assays [14]. The tumor suppressive functions of miR-193a-3p are widely described in the literature, and its expression is frequently decreased in solid tumors compared to normal tissues [15–18]. In addition, it has been shown that miR-193a-3p mimics induce apoptosis in many human cancer cell lines [18, 19]. Other phenotypes associated with ectopic miR-193a-3p expression include reduced migration and invasion, perturbed cell division, decreased angiogenesis, and increased reactive oxygen species (ROS) production [19–22]. Further, miR-193a-3p belongs to a subset of miRNAs which target several cell-cycle regulatory components, leading to a pronounced inhibition of cell proliferation [23]. The anti-cancer effects of miR-193a-3p are precluded by the repression of multiple target genes, including cancer related genes such as *CCND1*, *KRAS*, *RASSF1*, *STMN1*, and *MCL1* [19, 21, 24–26]. Despite the significant literature on the role of miR-193a-3p in tumor development, and evidence that miR-193a-3p mimics reduce cancer growth, further studies with an efficient miR-193a-3p mimic delivery system are required to elucidate the possible use of miR-193a-3p mimics as a novel therapeutic intervention for cancer.

1B3 is a synthetic chemically modified miR-193a-3p mimic with 100% sequence homology to the mature miR-193a-3p guide (antisense) strand and a fully complementary passenger (sense) strand. The molecule incorporates chemical modifications on its passenger strand to improve strand-selection and prevent inflammatory pathway activation *in vivo*. To overcome the challenge of delivering 1B3 to tumors upon systemic administration in experimental tumor models, it has been formulated in a novel lipid nanoparticle (INT-1B3). We recently evaluated the pharmacodynamic and pharmacokinetic profile of INT-1B3 and showed that it is efficiently delivered to tumor tissues and has a favorable safety profile (manuscript in preparation). In addition, RNA-sequencing and subsequent pathway analysis in a panel of cell lines confirmed the multi-targeted nature of 1B3 by revealing hundreds of target genes with key roles in tumor cell survival, proliferation, and migration [27]. In the current paper, we extensively document the multi-modal downstream effects of 1B3 in cell-based assays in a panel of tumor cell lines, and further investigate the mechanisms driving the anti-cancer effect of 1B3 *in vitro* by silencing different 1B3 target genes. We also confirm the anti-tumor activity of INT-1B3 in experimental human tumor-bearing mouse models, indicating its potential as promising novel modality for therapeutic intervention in oncology.

## RESULTS

### 1B3 suppresses human cancer cell growth through targeting multiple mechanisms and decreases cell migratory ability

To characterize the effect of 1B3 in cell-based assays, 1B3 was transiently transfected in a panel of human tumor cell lines from six different cancer types including colon (HCT116), liver (Hep3B, HUH-7, SNU-449), non-small cell lung carcinoma (A549, H460, H1299, H1975), skin (A2058), pancreatic (PANC-1), and breast (BT-549). The selected cancer cell lines had a variety of genetic backgrounds including *TP53* deletion or loss of function (Hep3B, SNU-449, H1299, H1975, A2058, PANC-1, and BT-549) and *KRAS* activating mutations (HCT116, A549, H460, and PANC-1). The level of cell-associated oligonucleotide (1B3) measured after transfection with 10-nM 1B3 was 100- to 1,000-fold higher compared to mock-transfected cells (Supplementary Figure 1), demonstrating significant transfection efficacy. As indicated in the Materials & Methods section, the 2-tailed RT-qPCR protocol detects both transfected 1B3 and endogenous miR-193a-3p. Notably, the increase after 1B3 transfection significantly exceeds the differences in basal expression among the tested cell lines. Further, 1B3 overexpression downregulated expression of the 1B3 target cyclin D1 (*CCND1*) by ~50% at the mRNA level (Supplementary Figure 2) and completely abolished expression at the protein level (Supplementary Figure 3), strongly demonstrating not only drug uptake, but efficient target engagement. These observed effects were 1B3-specific, as transfection of ‘negative’ miRNA control (3A1) did not lead to any significant changes in *CCND1* mRNA or CCND1 protein expression. 3A1 is based on a commercially available random sequence miRNA that has been validated to not produce identifiable biological effects and is routinely used as a ‘negative’ miRNA control.

### Cell count

**Figure 1:**
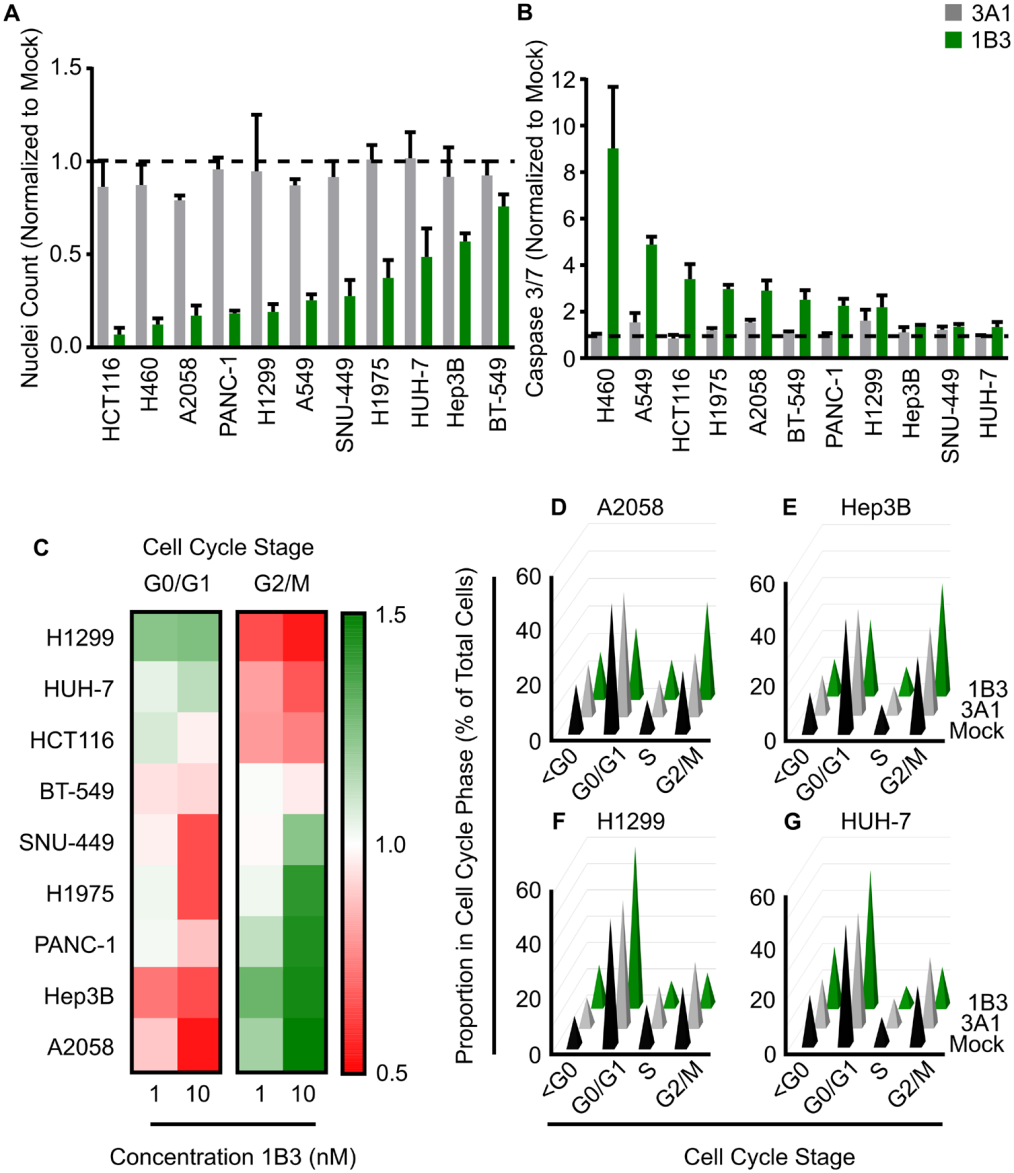
Effect of 1B3 on cell proliferation, apoptosis, and cell cycle in a panel of human cancer cell lines. Cells were transfected with either 1B3 or ‘negative’ miRNA control (3A1) in the presence of RNAiMAX transfection reagent. Non-transfected cells (mock) were also included to define baseline readout. (**A**) Nuclei count was determined 96 h after transfection with 10-nM of 1B3 by staining with Hoeschst-33342 and imaging using the Thermo CellInsite Automated Imager. Values were normalized to mock transfected cells. The dashed line represents the mock value of 1. (**B**) Caspase activation was measured at 48 or 72 h after transfection with 10-nM of 1B3 using Caspase-Glo 3/7 assay (Promega). Values were normalized to mock transfected cells. The time point with the highest caspase activation is shown. The dashed line represents the mock value of 1. (**C**) Cell cycle was assayed at 48, 72 or 96 h after transfection with 10-nM of either 3A1 or 1B3, and image-based DNA content analysis used to determine the proportion in each phase of the cell cycle. Values were normalized to mock transfected cells and the optimal time point shown. Proportion in each cell cycle phase for A2058 (at 72 h; **D**), Hep3B (at 48 h; **E**), H1299 (at 48 h; **F**), and HUH-7 (at 72 h; **G**) after transfection with 10-nM of either mock, 3A1 or 1B3. All values are the mean of at least three independent replicates. Error bars show standard deviation.

Overexpression of 1B3 reduced cell number in every cell line by 25–93% compared to the mock control 96 h after transfection (Figure 1A). The ‘negative’ miRNA control (3A1) was included, and its effect was similar to mock, strengthening the 1B3-specific effect. In five of the cell lines tested (HCT116, H460, A2058, PANC-1, and H1299), cell number was decreased by more than 80% upon transfection with 1B3. Only Hep3B and BT-549 had less than 50% reduction in nuclei count. Interestingly, the effect was dose-dependent as 1-nM 1B3 induced a milder reduction in cell number than 10-nM (Supplementary Figure 4). These data show that in a diverse range of genetic backgrounds and tissue types, 1B3 consistently reduces cell number.

### Apoptosis

As several biological processes can contribute to reduced cell number, including cell death, the effect of 1B3 on caspase 3/7 mediated apoptosis was investigated. Overexpression of 1B3 induced caspase 3/7 activity by more than 2-fold compared to the mock control in eight of the 11 cell lines tested (Figure 1B). The effect of the ‘negative’ miRNA control (3A1) was similar to mock, strengthening the 1B3-specific effect. The two *KRAS* wild type, *TP53* mutant, lung carcinoma cells (H460 and A549) had a very high caspase activation of 9- and 4.5-fold, respectively. Conversely, 1B3 induced only a mild caspase 3/7 activation (~1.3-fold) in the hepatocellular carcinoma (HCC) cancer cell lines SNU-449, HUH-7, and Hep3B. As with the effect on nuclei count, a milder effect was observed after treatment with 1-nM compared to 10-nM 1B3 (Supplementary Figure 5). To validate the effect of 1B3 on apoptosis, 1B3 was overexpressed in the two cell lines with the highest caspase 3/7 activation (H460, A549) and the two cell lines with the lowest caspase 3/7 activation (SNU449, HUH-7), and the level of cleaved poly (ADP-ribose) polymerase (PARP) was measured by Western blot. As expected, A549 and H460 had a strong increase in cleaved PARP compared to mock and 3A1-transfected cells, while no cleaved PARP was detected in SNU-449 or HUH-7 cells (Supplementary Figure 6). Additionally, the presence of apoptotic cells after 1B3 overexpression in A549 and H460 cells was confirmed by flow cytometric analysis of annexin V/PI-stained cells (data not shown). These data show that 1B3 causes apoptosis in some, but not all, of the cancer cell lines tested, indicating that it is one of the anti-proliferative mechanisms of 1B3.

### Cell cycle arrest

Premature cell cycle arrest can also reduce cell number over time, therefore the effect of 1B3 on the cell cycle was investigated using image based DNA-content analysis [28]. As shown in Figure 1C, 1B3 treatment induced a cell cycle arrest compared to mock transfected cells in all cell lines except BT-549. The effect of 3A1 was similar to mock (Supplementary Figure 7) strengthening the 1B3-specific effect. In three cell lines (HCT116, H1299, and HUH-7) the proportion of G0/G1 cells increased after 1B3 treatment, while in the other five cell lines (SNU-449, Hep3B, H1975, PANC-1, and A2058) the proportion in G2/M increased (Figure 1C). A2058 and Hep3B cells are depicted as cell lines with the strongest arrest in the G2/M phase after 1B3 treatment, compared to mock and 3A1 transfected cells (Figure 1D and 1E), while H1299 and Huh-7 cells had the strongest arrest in G0/G1 (Figure 1F and 1G). As expected, this effect was dose-dependent for all cell lines except HCT-116.

### Senescence

**Figure 2:**
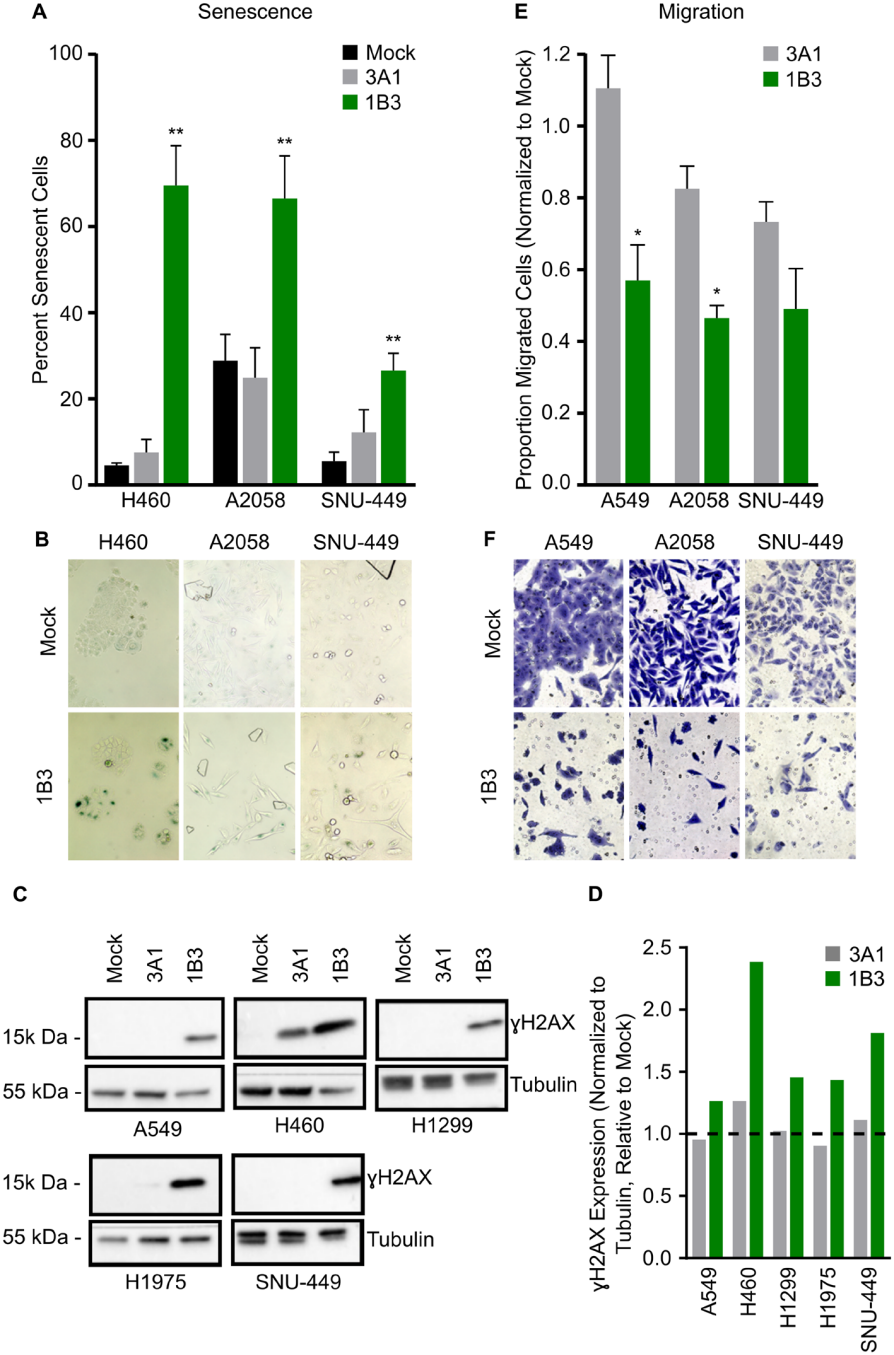
Effect of 1B3 on tumor cell senescence, migration, and DNA damage. Cells were transfected with either 1B3 or ‘negative’ miRNA control (3A1) in the presence of RNAiMAX transfection reagent. Non-transfected cells (mock) were included to define baseline readout. (**A**) Senescence was assayed using the SA-β galactosidase assay (Cell Signaling) four days after transfection with 10-nM of either 1B3 or 3A1, and 500 cells in total counted for each condition. The proportion of senescent cells relative to non-senescent cells was calculated. Error bars show the standard deviation of three biological replicates. ^**^ indicates *p* < 0.01 calculated by Dunnett’s multiple comparison test. Representative images of senescence after transfection with 1-nM 1B3 (**B**) were captured at 10x magnification. (**C**) Cell lysates for Western blotting for ɣ-H2AX were harvested 72 h after transfection with 10-nM of either 1B3 or 3A1. Ten to 20 μg protein was separated by SDS-PAGE, transferred to PVDF membranes, and hybridized with ɣ-H2AX and tubulin antibodies. Tubulin was used as protein loading control. Band intensities were quantified by densitometry using ImageJ software (**D**) and values normalized to the tubulin loading control and are shown relative to mock transfected cells. The dashed line represents the mock value of 1. (**E**) Transwell cell migration was assayed 48 h after transfection with 10-nM or either 1B3 or 3A1. Ten images per condition at 20x magnification were counted. Values were corrected for the effect of 1B3 on viability using counts from a concurrent 24-well cell culture plate. Values were normalized to mock (mock = 1). Error bars show the standard deviation of two independent replicates. ^*^ indicates *p* < 0.05 calculated by two-tailed *t*-test. Representative images of migration after transfection with 10-nM 1B3 (**F**) were captured at 20x magnification.

Because 1B3 overexpression reduced cell number but did not always lead to a strong caspase 3/7 induction (apoptosis), the effect of 1B3 on cell senescence (type of growth arrest that primarily occurs in aged cells) was investigated in a subset of cell lines. The cell lines selected represent a range of apoptotic response to 1B3 including SNU-449, Hep3B, A2058, and H460. To ensure that the effect of 1B3 on cell count did not bias the results, a total of 500 cells was counted for each condition. Overexpression of 1B3 increased the number of cells expressing the senescence marker, SA-β-gal, compared to mock and 3A1-transfected cells in all cell lines tested (Figure 2A). A representative image shows an increase in SA-β-gal-stained cells in 1B3-treated cells compared to mock-transfected cells (Figure 2B). 1B3 had the strongest effect in H460 cells (16-fold induction), while a 5- and 2.3- fold increase was observed in SNU-449 and A2058 cells, respectively. In addition, 1B3 transfection induced a 1.8-fold increase in senescence in Hep3B cells (Supplementary Figure 8). Collectively, evidence in four cell lines suggests that 1B3 overexpression induces cell senescence.

### DNA damage

Both G2/M cell cycle arrest and cell senescence are associated with an accumulation of DNA replication errors [29], therefore the presence of the DNA damage marker ɣH2AX (histone variant that is phosphorylated in response to double-strand break DNA damage) was investigated in a subset of 1B3-treated cells. Western blot analysis confirmed that ɣH2AX expression was increased in 1B3-transfected cells relative to both mock and 3A1-transfected cells in all cell lines tested (Figure 2C and 2D). This included the G2/M arrested cells (H1975 and SNU-449), but also to a milder extend in the G0/G1 arrested cell line H1299. Moreover, ɣH2AX was increased in both highly apoptotic cell lines (A549 and H460). These data suggest that 1B3 specifically enhances DNA damage in treated cells, regardless of the cell cycle arrest phase.

### Migration

Finally, the effect of 1B3 on cell migration was investigated in a range of cancer cell types including A549, A2058, and SNU-449. The strongest effect on transwell cell migration was observed in A2058 and SNU-449 cells (54 and 51% decrease, respectively), while a 43% decrease was observed in A549 cells when compared to mock (Figure 2E). Although 3A1 slightly reduced migration in A2058 and SNU-449 cells (18% and 27%, respectively), the effect of 1B3 was much more pronounced. To ensure that the effect of 1B3 on cell count did not bias the results, a correction factor was applied (as indicated in Materials and Methods section). Figure 2F shows a representative image of migrated cells after 1B3 or mock transfection.

Taken together, these data shows that transfection of 1B3 induces a range of anti-cancer phenotypes in a panel of cancer cell lines, supporting its potential as a novel cancer therapeutic by targeting multiple mechanisms (Table 1).

**Table 1:** Effect of 1B3 on cell proliferation, apoptosis, cell cycle, senescence, and migration in a panel of human cancer cell lines

Cell line	Cell count	Caspase 3/7	Cell Cycle	Senescence	DNA damage	Migration
HCT116	✓✓✓	✓✓✓	G0/G1 arrest	-	-	-
H460	✓✓✓	✓✓✓	-	✓✓✓	✓✓	-
A2058	✓✓✓	✓✓	G2/M arrest	✓✓	-	✓✓
PANC-1	✓✓✓	✓✓	G2/M arrest	-	-	-
H1299	✓✓✓	✓✓	G0/G1 arrest	-	✓	-
A549	✓✓	✓✓✓	-	-	✓	✓
SNU-449	✓✓	✓	G2/M arrest	✓✓✓	✓	✓✓
H1975	✓✓	✓✓	G2/M arrest	-	✓	-
HUH-7	✓✓	✓	G0/G1 arrest	-	-	-
Hep3B	✓	✓	G2/M arrest	✓	-	-
BT-549	✓	✓✓	✘	-	-	-

✓✓✓ = > 3-fold increase or > 75% decrease, relative to mock, ✓✓ = 2 to 3-fold increase or 50–75% decrease, relative to mock, ✓ = 1.25 to 2-fold increase or 20–50% decrease, relative to mock, ✘ = no effect of 1B3 in the cell line tested, - was not tested in this assay.

### 1B3 phenotype can be partially phenocopied by individual or combined siRNA

To further investigate the mode of action through which 1B3 exerts its effects on tumor cells, selected individual 1B3 target genes were silenced using siRNAs, and their effect on key phenotypes measured. MiRNAs suppress the expression of numerous different target mRNAs. We therefore tested siRNAs against several known and validated 1B3 mRNA targets which have been documented as critical for its tumor suppressive functions. These include regulators of cell cycle (*CCND1*, *CDK6*), proliferation (*KRAS*), apoptosis (*MCL1*, *YWHAZ*), and cytoskeletal organization (*STMN1*). As the effects of 1B3 varied depending on cellular context, the effects were investigated in several human cancer cell lines representing three different cancer types.

**Figure 3:**
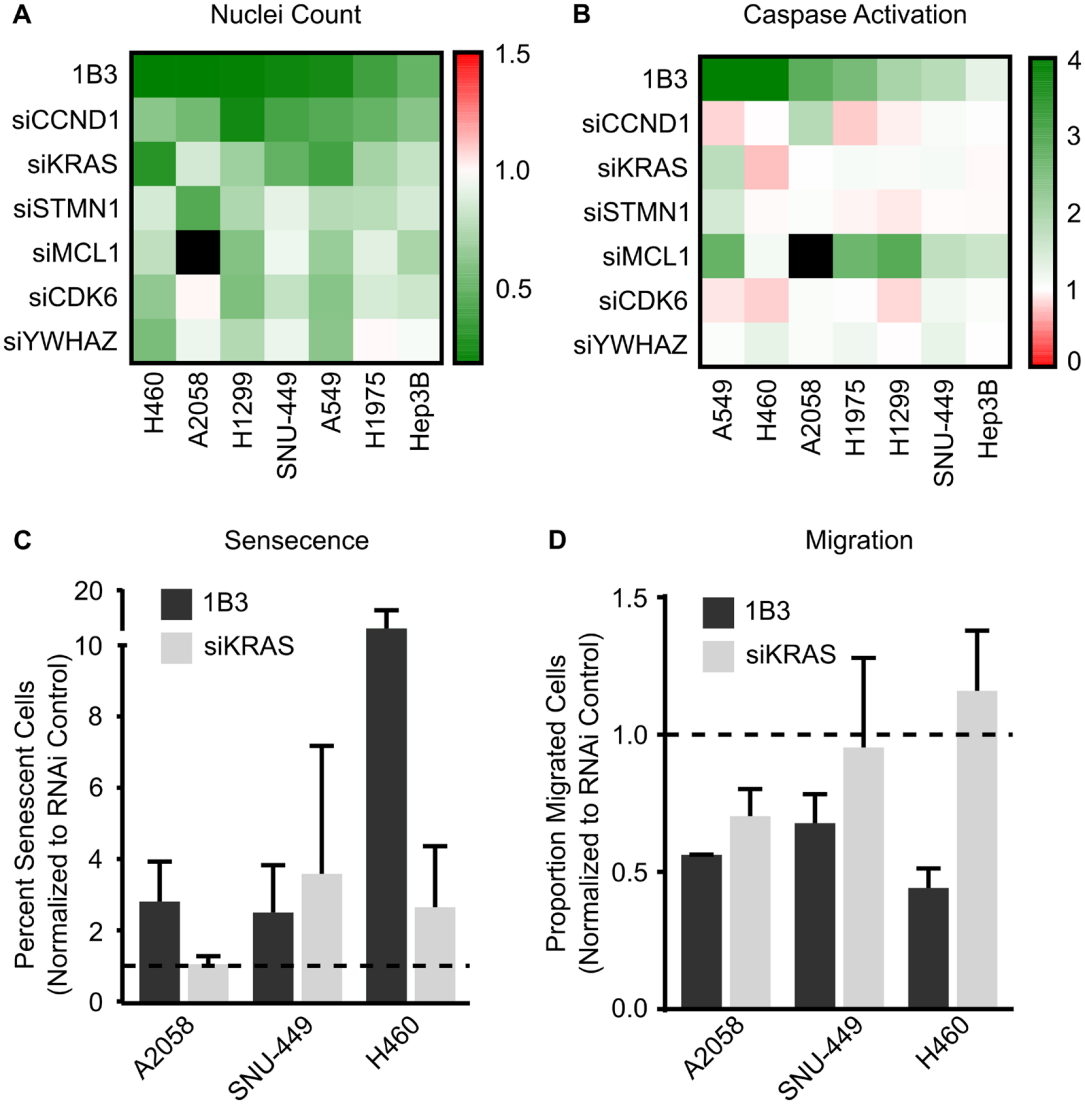
Effect of silencing individual 1B3 target gene expression on cell proliferation, apoptosis, senescence, and migration in a panel of human cancer cell lines. Cells were transfected with 10-nM of either 1B3, ‘negative’ miRNA control (3A1), negative control siRNA (siPOOL), or individual siRNAs in the presence of RNAiMAX transfection reagent. Non-transfected cells (mock) were also included to define baseline readout. (**A**) Nuclei count was determined after 96 h by staining with Hoeschst 33342 and imaging using the Thermo CellInsite Automated Imager. Values were normalized to mock transfected cells and represent the mean of three independent replicates. Black squares indicate combinations that were not tested. (**B**) Caspase activation was measured 48 or 72 h after transfection using Caspase-Glo 3/7 assay (Promega). Values were normalized to mock transfected cells and represent the mean of three independent replicates. The time point with the highest caspase activation is shown. Black squares indicate combinations that were not tested. (**C**) Senescence was assayed using the SA-β-galactosidase assay (Cell Signaling) four days after transfection and 500 cells in total counted for each condition. The proportion of senescent cells relative to non-senescent cells was calculated. 1B3 values were normalized to 3A1, and siKRAS values normalized to siPOOL. Error bars show the standard deviation of at least two independent replicates. Statistical significance was not determined. Dashed lines represent the mock value of 1. (**D**) Transwell cell migration was assayed 48 h after transfection. Ten images per condition at 20x magnification were counted. Values were corrected for the effect of 1B3 on viability using counts from a concurrent 24-well plate. 1B3 values were normalized to 3A1, and siKRAS values normalized to siPOOL. Error bars show the standard deviation of at least two independent replicates, statistical significance was not determined. Dashed lines represent the mock value of 1.

Selected siRNAs were overexpressed in a maximum of seven cancer cell lines and the effect on reduction in cell number (Figure 3A), induction of apoptosis (Figure 3B), induction of cell cycle arrest (data summarized in Supplementary Table 1), induction of cellular senescence (Figure 3C), and inhibition of migration (Figure 3D) compared to that of 1B3. The negative siRNA control (siPOOL) was included, and its effect was similar to mock (Supplementary Figure 9A and 9B), strongly suggesting a selective siRNA-specific effect. In general, siRNAs reduced cell count to a varied extent in the seven cell lines (Figure 3A). No siRNA was able to mimic the exact effect of 1B3 in all cell lines, although some were able to closely phenocopy the 1B3 effect in several. For example, siCCND1, siKRAS, and siSTMN1 reduced cell number by a similar extent to 1B3, but only in some contexts. SiCCND1 had the most consistent effect on cell count, reducing cell number by more than 50% in three different cell lines (H1299, SNU-449, and A549) although the effect of 1B3 was even stronger when used in similar conditions. Ultimately the effect of the siRNAs on cell count (if observed) were milder than the effect of 1B3.

Additionally, the contribution of selected 1B3 targets to apoptosis was measured with a caspase 3/7 activation assay. In general, no siRNA mimicked the exact effect of 1B3 on apoptosis in all cell lines. Nevertheless, siMCL1 closely phenocopied the effect of 1B3 in five out of the seven cell lines tested (A549, H1975, H1299, SNU-449, and Hep3B).

Furthermore, the siRNAs tested had a diverse effect on cell cycle arrest in five different cell lines (data summarized in Supplementary Table 1). Again, no siRNA mimicked the exact effect of 1B3 in all cell lines. For example, siCCND1, siCDK6, siMCL1, and siYWHAZ phenocopied the effect of 1B3 by inducing a G0/G1 arrest, but only in one cell line (H1299). Nonetheless, and consistent with the effect of 1B3, siSTMN1 induced a G2/M arrest in three cell lines (Hep3B, SNU-449, and A2058). These data suggest that although some of the 1B3 targets tested play a role in cell cycle arrest, this effect is cell line-dependent and does not always replicate the effect of 1B3.

Next, the contribution of 1B3 targets *KRAS* and *MCL1* to induction of cellular senescence was measured. Neither siRNA mimicked the exact effect of 1B3 in all cell lines tested (A2058, SNU-449, and H460) (Figure 3C). Although siKRAS closely phenocopied the effect of 1B3 in SNU-449, it only partially copied this effect in H460 (a 2-fold increase by siKRAS *versus* the 11-fold increase by 1B3). siMCL1 on the other hand did not induce senescence in any of the cell lines (data not shown). This shows that in some contexts, silencing of *KRAS* may be responsible for 1B3 induced senescence, although this effect was not observed in all cell lines.

Finally, the role of 1B3 targets *KRAS* and *STMN1* in reduction of migration was assessed (Figure 3D). While siKRAS closely mimicked the effect of 1B3 on reducing A2058 cell migration, it showed no impact in SNU-449 and A549 cells. Further and in contrast to 1B3, siSTMN1 did not reduce cell migration in any of the cell lines tested (A2058, SNU-449, and A549) (data not shown). Once again, this suggests that *KRAS* repression may contribute to the effect of 1B3 on migration in some contexts, but not in all cell lines.

**Figure 4:**
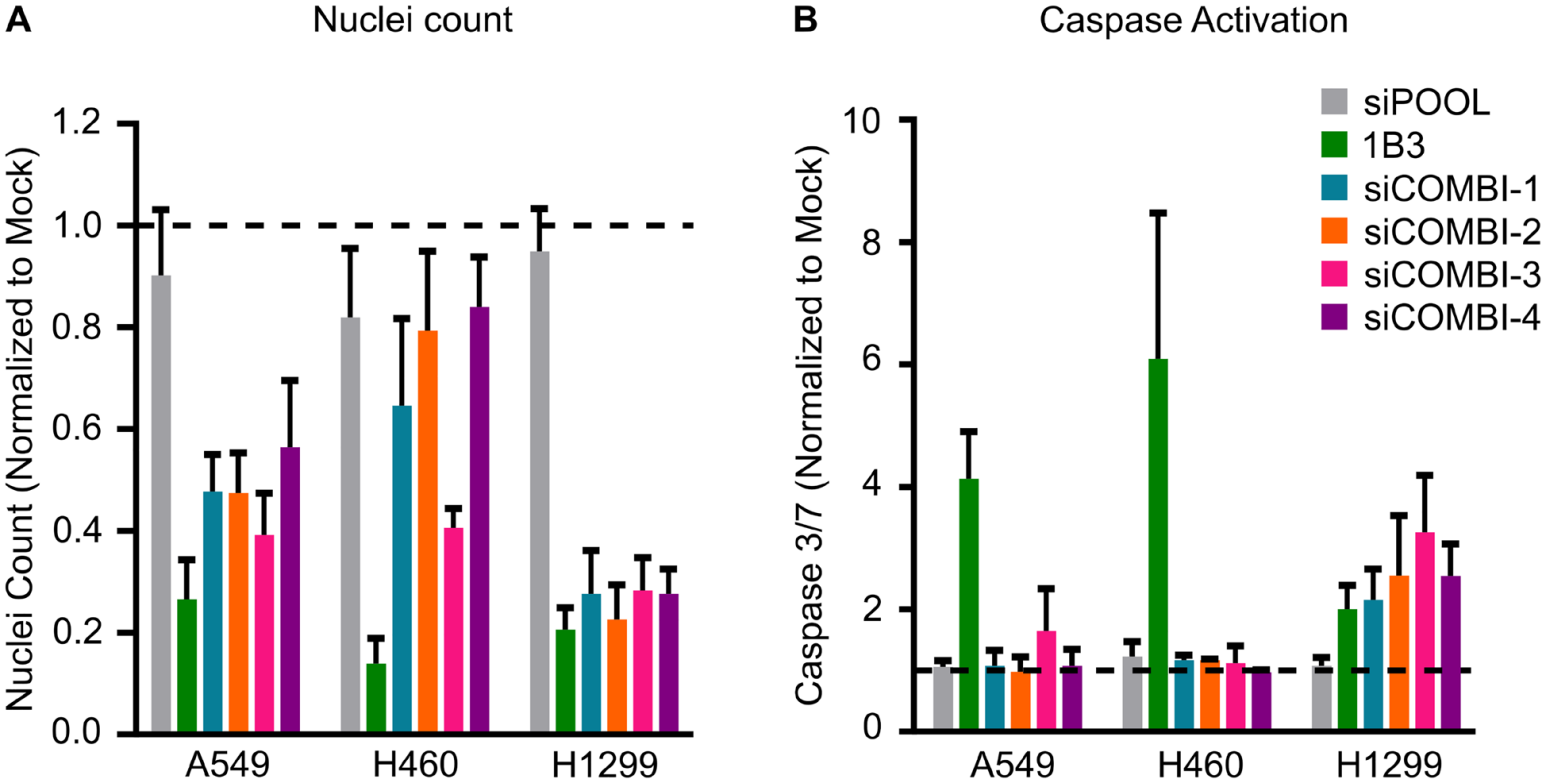
Effect of silencing combinations of 1B3 target gene expression on cell proliferation and apoptosis in selected human cancer cell lines. Cells were transfected with 10-nM of either 1B3, ‘negative’ miRNA control (3A1), negative control siRNA (siPOOL), or a combination of siRNAs with a total RNAi concentration of 9–14 nM (see Table 3 for compositions). Non-transfected cells (mock) were also included to define baseline readout. (**A**) Nuclei count was determined after 96 h by staining with Hoeschst 33342 and imaging using the Thermo CellInsite Automated Imager. Values were normalized to mock transfected cells. (**B**) Caspase activation was measured 48 or 72 h after transfection using Caspase-Glo 3/7 assay (Promega). Values were normalized to mock transfected cells. The time point with the highest caspase activation is shown. All values are the mean of at least three independent replicates. The dashed line represents the mock value of 1.

Together, after silencing several different individual 1B3 target genes in a range of human cancer cell lines, it is evident that some targets contribute to some of the phenotypes in a cell line-dependent manner. However, no single target gene impacted all the phenotypes induced by 1B3 overexpression (Table 2). Because a miRNA simultaneously reduces the expression of numerous target genes, we hypothesized that artificially silencing multiple 1B3 targets *in vitro* might more closely phenocopy the effect of 1B3 than individual siRNA. To address this, we selected three human cancer cell lines (A549, H460, and H1299) and transfected them with combinations of siRNAs, then measured the effect on cell count (Figure 4A) and caspase 3/7 activity (Figure 4B). As siCCND1 consistently reduced cell count when used individually, and siMCL1 most consistently induced caspase 3/7 activation, these were included in all combinations along with other selected siRNAs (see Table 3 for siCOMBI compositions). To avoid toxicity from overloading the cells with siRNAs, each siRNA was used at a concentration of 2–3 nM, to achieve a total siRNA concentration of 9–14 nM, in line with the concentration of 1B3 used. Consequently, the effect of siRNA combinations on target gene expression was slightly milder than the effect of individual siRNA, although target genes were always reduced by more than 50%, similar to the effect of 1B3 (Supplementary Figure 10). Not all combinations were able to phenocopy the 1B3 effect in all tested cell lines. For example, all combinations in H1299, all except siCOMBI-4 (siCCNC1, siMCL1, and siSTMN1) in A549, and only siCOMBI-3 (siCCND1, siMCL1 and siKRAS) in H460, closely phenocopied the effect of 1B3 by reducing cell viability by more than 50% (Figure 4A). Surprisingly, none of the combinations induced caspase 3/7 activation in A549 or H460 cells, despite a very strong effect of 1B3 in these cell lines (Figure 4B). On the other hand, and similar to 1B3, in H1299 cells all siRNA combinations induced caspase 3/7 by more than 2-fold. These data suggest that even silencing several 1B3 target genes does not fully reproduce the phenotypic effect of 1B3 overexpression in multiple cell lines.

**Table 2:** Effect of silencing 1B3 targets with siRNA on several cell phenotypes

Target gene	Cell count (7)	Caspase 3/7 (7)	Cell Cycle (5)	Migration (3)	Senescence (3)
*CCND1*	✓✓✓	✘	✓✓	-	-
*KRAS*	✓✓	✘	✓	✓	✓✓
*MCL1*	✘	✓✓✓	✘	-	✘
*STMN1*	✓	✘	✓	✘	-
*YWHAZ*	✘	✘	✓	-	-
*CDK6*	✘	✘	✓	-	-

✓ = phenocopies 1B3 effect in 1 cell line, ✓✓ = phenocopies the effect of 1B3 in 2 cell lines, ✓✓✓ = phenocopies the effect of 1B3 in 3 or more cell lines, ✘ = does not phenocopy 1B3 effect in any cell line tested, - was not tested in this assay. Numbers in brackets indicate the number of cell lines assessed for each phenotype.

**Table 3:** Composition of siRNA combinations

	siCCND1	siMCL1	siCDK4	siCDK6	siKRAS	siSTMN1	siYWHAZ
siCOMBI-1	2 nM	2 nM	2 nM	2 nM	2 nM	2 nM	2 nM
siCOMBI-2	3 nM	3 nM	3 nM	3 nM			
siCOMBI-3	3 nM	3 nM			3 nM		
siCOMBI-4	3 nM	3 nM				3 nM	

### 1B3 inhibits tumor growth *in vivo*

Small double-stranded oligonucleotides targeting mRNA expression, such as siRNAs or miRNAs, have promising therapeutic applications in the treatment of human diseases. However, such unmodified oligonucleotides are easily degraded by nucleases and have low membrane permeability due to their high molecular weight and negative charges. Hence, an efficient drug delivery system is needed to improve their therapeutic efficacy [30]. Therefore, to evaluate the efficacy of 1B3 on experimental tumor models, a novel lipid nanoparticle-based formulation was developed for the efficient delivery of functional 1B3 (INT-1B3; see Materials and Methods section) in target organs (manuscript in preparation).

**Figure 5:**
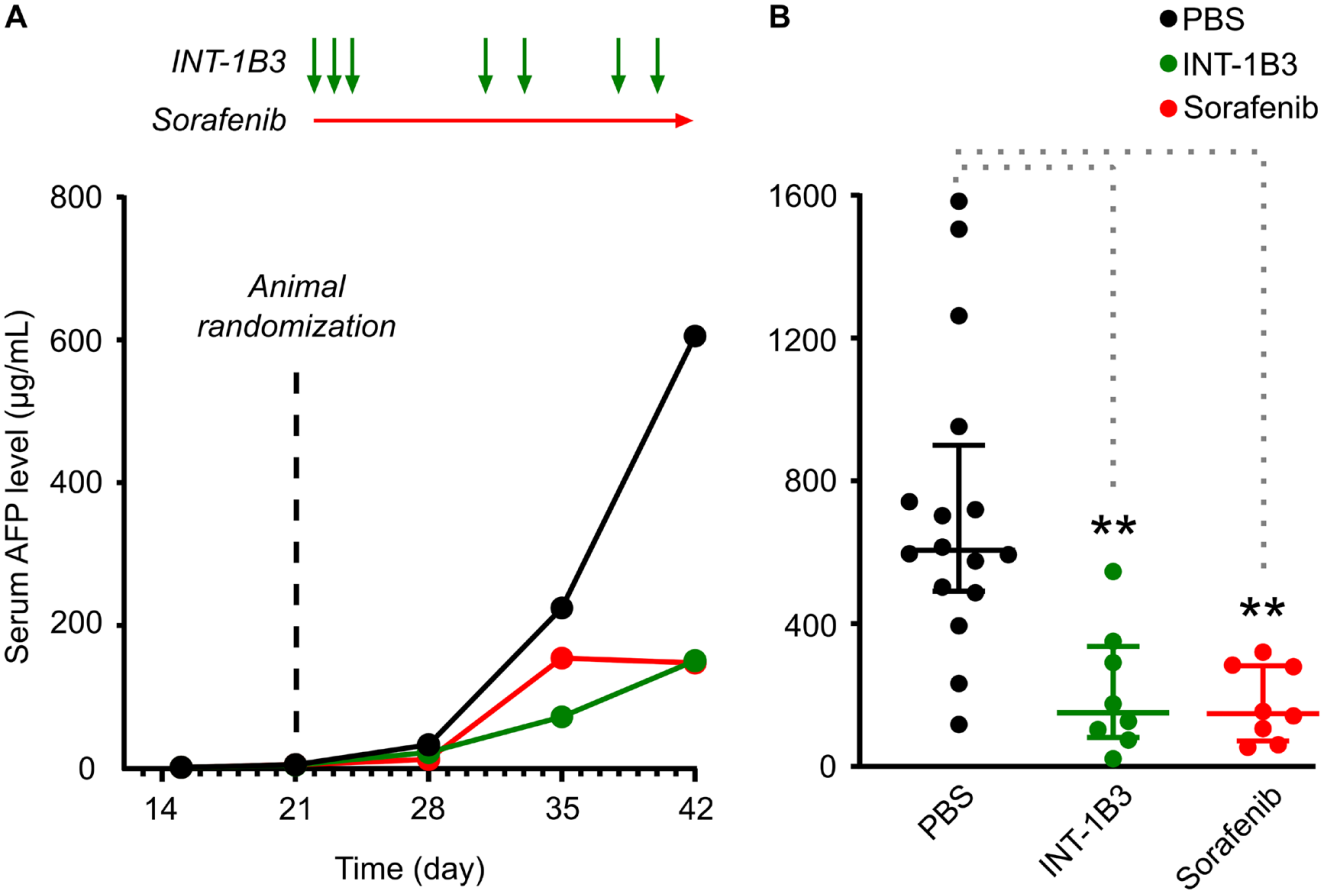
Effect of INT-1B3 on orthotopic human HCC Hep3B tumor growth in immune-compromised mice. Human HCC Hep3B tumor fragments were implanted into the left liver lobe of SCID/Beige mice (orthotopic tumor model), as indicated in the Materials and Methods section. Serum AFP levels were measured as indicator of liver tumor development. On day 21, animals were randomized in experimental groups based on increasing serum AFP levels (median = 5.2 μg/mL), and treatment with test items started one day after (i.e., on Day 22). Animals were treated with either vehicle (PBS, i.p.; black) or INT-1B3 (10 mg/kg/administration, i.v.; green) daily for three days during the first week, then twice weekly for the following two weeks. Animals were also treated with sorafenib (10 mg/kg/administration, p.o., BID; red) as reference control in this experimental tumor model. Serum AFP levels were determined weekly throughout the study. (**A**) Time-dependent evolution of serum AFP levels (median, expressed as μg/mL). See Supplementary Figure 12 for a semi-log scale representation of the ~3 order of magnitude increase in AFP levels throughout the study. (**B**) on Day 42, serum AFP values from individual animals, median (horizontal bar), and interquartile range (vertical error bar) are presented on linear scale for the indicated experimental groups. ^**^ indicates *p*-value < 0.01 by the 2-tailed non-parametric Mann–Whitney statistical analysis (*p* values of 0.005 and 0.002 for INT-1B3 (*n* = 8) and sorafenib (*n* = 8), respectively) as compared to PBS-treated group (*n* = 16).

To evaluate INT-1B3 in the experimental human HCC Hep3B tumor model, Hep3B tumor fragments were implanted into the left liver lobe (orthotopic tumor model) of SCID/Beige mice. After tumor establishment, animals were treated intravenously with 10 mg/kg/administration INT-1B3 or vehicle control (PBS) for three consecutive days in the first week and maintained on a twice weekly treatment schedule for an additional three weeks (Figure 5A). As it is technically difficult to monitor tumor volume over time in such an orthotopic model, plasma human alpha fetoprotein (AFP) was used as a surrogate measure of liver tumor development [31]. Tumor size (plasma AFP) was monitored weekly, until animals were euthanized according to humane endpoint criteria. AFP levels were confirmed as a sensitive readout for HCC tumor development after tumors were harvested and weighed on the day of sacrifice and a strong correlation (R^2^ = 0.706) was observed between individual animal plasma AFP levels and tumor weights (Supplementary Figure 11). Notably, INT-1B3 treatment resulted in a significant reduction in median AFP levels over time, highlighting its anti-tumor activity in the Hep3B model (Figure 5A). INT-1B3 treatment was initiated on established tumors with increasing AFP levels, although because levels increased by ~3 orders of magnitude throughout the study this is not apparent on a linear scale. An alternative representation on a semi-log scale confirms the increase of AFP levels during the first three weeks of the study (Supplementary Figure 12). The effect on tumor growth was further quantified by determining the T (treatment)/C (control) ratio (a measure of the extent of tumor growth inhibition) on day 42 post-tumor fragment implantation. Significantly, tumor growth was reduced by ~75% in INT-1B3-treated animals (*p* = 0.0005), reaching the same growth inhibition as the kinase inhibitor sorafenib (Figure 5B) used as positive control for this experimental tumor model. These data indicate potent and significant anti-tumor activity of INT-1B3 in the Hep3B model at a well-tolerated dose of 10 mg/kg. Importantly, drug delivery was illustrated by a significant 25-fold increase in 1B3 levels in tumors harvested on the day of sacrifice from INT-1B3-treated mice compared to the PBS control animals (Supplementary Figure 13A). In line with this, *CCND1* mRNA expression in these tumors decreased by ~50%, indicating that 1B3 is functional at a molecular level (target engagement) in the tumors (Supplementary Figure 13B).

**Figure 6:**
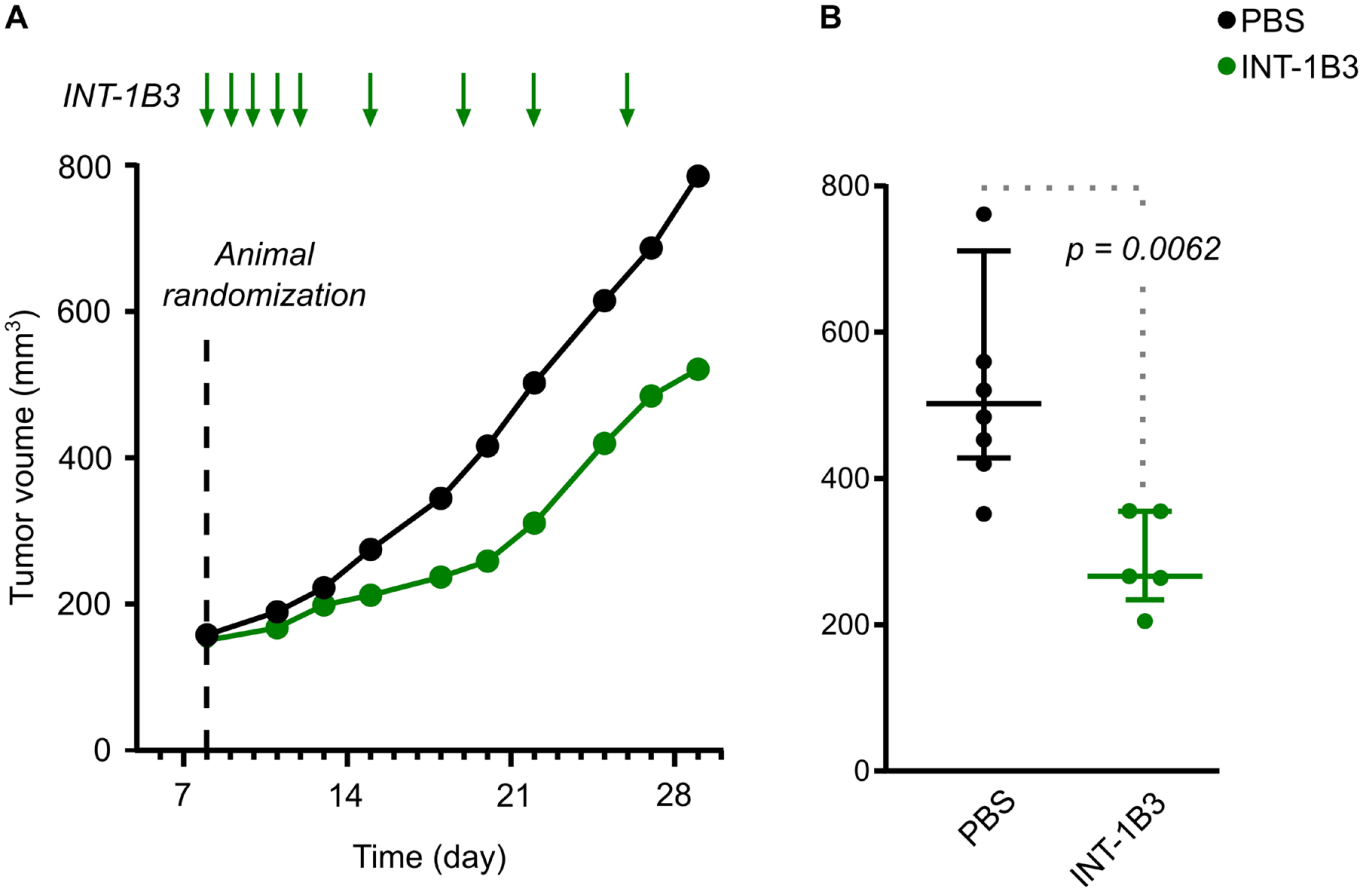
Effect of INT-1B3 on subcutaneous human melanoma A2058 tumor growth in immune-compromized mice. Human melanoma A2058 tumor cells (5 × 10^6^) in 0.1-mL PBS were injected subcutaneously in the flank BALBc/nude mice. After eight days, animals were randomized to experimental groups based on subcutaneous tumor size (median ~150 mm^3^; established subcutaneous tumor model). Animals were then treated with either vehicle (PBS, i.p.; black) or INT-1B3 (3 mg/kg/administration, i.v.; green) daily for five days the first week, then twice weekly for the next two weeks. Subcutaneous tumor volumes (expressed as mm^3^) were calculated throughout the study based on caliper-aided tumor measurements and applying the following formula: (length × width^2^)/2. (**A**) Time-dependent evolution of tumor volumes (median values expressed as mm^3^). (**B**) on Day 22, individual tumor volumes, median (horizontal bar) and interquartile range (vertical error bar), are presented for the indicated experimental groups. A 2-tailed non-parametric Mann–Whitney statistical analysis was performed for INT-1B3 (*p* = 0.0062) as compared to PBS-treated group, with *n* = 7 and 5 for PBS and INT-1B3 groups, respectively.

To extend these findings, efficacy of INT-1B3 on tumor growth was further investigated in the human melanoma A2058 subcutaneous tumor model. Once tumors reached a median volume of ~150 mm^3^ treatment with 3 mg/kg/administration INT-1B3 or vehicle control (PBS) was administered intravenously daily for five days during the first week then twice weekly for the next two weeks (Figure 6A). Tumor volume was monitored three times weekly throughout the study until the animals were euthanized according to humane endpoint criteria. Consistent with the effect observed in the Hep3B model, the median tumor volume in the INT-1B3 treatment group revealed a significant tumor growth delay compared to the PBS control (Figure 6A). The effect on tumor growth was further quantified by determining the T/C ratio on day 22 post-cell implantation which revealed 54% tumor growth inhibition in INT-1B3 treated animals relative to the PBS-treated animals (*p* = 0.0062) (Figure 6B). Four animals were sacrificed on day 15 (i.e., 48 h after the fifth INT-1B3 treatment) and tumors harvested for analysis of drug delivery and target engagement. Importantly, more than 1,500 ng 1B3 per gram of tumor was detected in tumors from INT-1B3 treated mice (Supplementary Figure 14A). Conversely, less than 1 ng 1B3 per gram of tumor was detected in tumors from the PBS-treated group, strongly suggesting low, if any, endogenous miR-193a-3p expression in this tumor model. Additionally, INT-1B3 treatment decreased tumor *CCND1* mRNA expression by 43% relative to PBS-treated tumors (Supplementary Figure 14B). This clearly indicates that 1B3 is effectively delivered and functional in experimental tumor models and leads to significant anti-tumor activity.

## DISCUSSION

Cancer utilizes an array of mechanisms to escape cell death and proliferate without control [2]. MiRNAs are naturally occurring small double-stranded RNA molecules which modulate gene expression during development and (patho)physiological conditions. MiRNAs silence multiple targets, and therefore multiple signaling pathways, presenting an exciting new class of potential cancer therapeutics [9, 10]. Here we provide a comprehensive biological profile of the synthetic miR-193a-3p mimic, 1B3, both *in vitro* and *in vivo*. 1B3 consistently reduced cancer cell proliferation, through a range of mechanisms, in a variety of cancer cell lines. This confirms our recent transcriptome-wide analysis of differential gene expression demonstrating 1B3 as a strong phosphatase and tensin homolog (PTEN) pathway activator which governs various cell processes including tumor cell survival, proliferation, and migration [27]. Our results support the role of miR-193a-3p as a tumor suppressor miRNA, and its further development as a clinically relevant cancer drug candidate. Further, we show that 1B3 functions through a diverse range of target genes which ensures a robust response across different genetic contexts.

The anti-proliferative effects of 1B3 *in vitro* are in agreement with data from a miR-193a-3p mimic screen in a panel of 122 human cancer cell lines from 13 different cancer types [23]. In that study, miR-193a-3p overexpression reduced cell number in 112 of the 122 cell lines. Moreover, in parallel screens, miR-193a-3p overexpression induced caspase activation by more than 2-fold in half the cell lines, and induced senescence by more than 2-fold in one third of cell lines [23]. In line with these findings, we showed that increased 1B3 levels reduced proliferation in all 11 cell lines tested and activated caspase 3/7 in eight of them. Importantly, the three cell lines in which 1B3 did not strongly induce caspase 3/7 (SNU449, HUH-7, and Hep3B) still had a significant decrease in cell number. Although these three cell lines are all liver cancer cell lines, other groups have shown that miR-193a-3p acts as a tumor suppressor miRNA in liver cancer [32, 33] and we observed a strong tumor growth inhibition in the human HCC Hep3B orthotopic tumor model. This indicates that 1B3 reduces proliferation through different mechanisms in different cell lines and cancer types. To that end, 1B3 increased senescence and induced a G2/M cell cycle arrest in SNU449 and Hep3B, both mechanisms which can contribute to reduced cell growth.

MiR-193a-3p has been described as a cell-cycle regulating miRNA due to its silencing of several important cell cycle regulators, including *CCND1*, *CCND2*, *CCNE2*, *CDK2*, *CDK4*, and *CDK6* [23], which was further confirmed in our recent RNA sequencing based study [27]. As expected, 1B3 strongly affected cell cycle progression, with a cell growth arrest observed in eight out of the nine cell lines tested. Intriguingly, in five of the cell lines, cells accumulated in the G2/M phase, while another three cell lines accumulated in the G0/G1 phase. Arrest in both phases after miR-193a-3p transfection have been described previously—Liu et al. [34] demonstrated a G0/G1 arrest in prostate cancer cells while Pruikkonen et al. [21] observed an increase in mitotic ovarian cancer cells. Interestingly, cell lines arrested in the G2/M phase lack functional expression of the DNA damage repair-associated protein TP53 (SNU449, Hep3B, H1975, PANC-1, and A2058), while two of the three arrested in G0/G1 express (HCT116) or overexpress (HUH-7) this protein. Further to the cell cycle arrest, 1B3 overexpression increased levels of the DNA damage marker ɣH2AX. Thus, 1B3 induced DNA damage may require functional TP53 for repair, and in cell lines lacking *TP53* expression deficient repair could lead to further DNA damage accumulation and G2/M arrest. Nevertheless, the cell cycle is controlled by several factors and further investigation is required to dissect the specific role of 1B3 on this phenotype in different genetic contexts.

An important observation is that some effects of 1B3 differ between human cancer cell lines. As illustrated by the different cell cycle arrest patterns and caspase 3/7 activation, the genetic background of the cancer cell may influence the exact phenotype induced by 1B3 overexpression. Despite different upstream events, 1B3 overexpression reduced proliferation in every cell line, regardless of genetic background. For example, the *KRAS* mutant cell lines HCT116, H460, A549, and PANC-1 cells were among those with the strongest response to 1B3, but proliferation was also reduced in wild-type *KRAS* expressing cells such as A2058, H1299, and SNU449. Expression of oncogenic *KRAS* is linked to aggressive disease and therapeutic resistance, yet despite years of effort no effective inhibitor has reached the clinic [35, 36]. Consequently, 1B3 may be a useful therapy for *KRAS*-driven tumors, but also for tumors with wild-type expression of this oncogene. This broad application of 1B3 in different oncogene-driven tumors is supported by the 50% tumor growth inhibition of *BRAF*-mutated A2058-derived tumors after INT-1B3 treatment *in vivo*.

The exact mechanism through which miR-193a-3p induces an anti-cancer effect has been widely studied and although it is almost universally described as a tumor-suppressor miRNA, the genes which it targets are not always consistent. While some studies connect the anti-proliferative effect of miR-193a-3p mimics to a single gene target, our data suggests this effect is the result of unique, simultaneous, regulation of multiple mRNAs. In support of this, our recent transcriptome wide analysis of six human cancer cell lines uncovered activation of the PTEN tumor suppressor pathway as one of several key mechanisms behind the 1B3 mode of action [27]. 1B3 exerts its multi-modal effect, at least in part, by targeting critical genes in the PTEN pathway which reduces cell proliferation and migration. Because miRNAs only need a short sequence of complementary bases to repress gene expression, each miRNA can bind to hundreds of targets in the same or different pathways, inducing a cascade of genetic changes within a cell [5, 37]. More than 50 different targets of miR-193a-3p have been described, including *CCND1*, *cKIT*, *HMGB1*, *GRB7*, *PLAU*, *RASSF1*, *PAK4*, *S6K2*, *STMN1*, and *PTP1B* [16, 21, 24, 26, 33, 38–43]. In this study, we tested a selection of only six siRNA to silence miR-193a-3p targets (including several important in the PTEN pathway), so it is possible we did not investigate the one target of miR-193a-3p that affects all phenotypes in all cell lines. It is more likely, however, that 1B3 function is determined by simultaneous regulation of several targets, and that the precise effect depends on the genetic background of the cancer cell. Large scale sequencing of miRNA-mRNA interactions revealed that each miRNA has a unique ‘targetome’ which varies between cell types [44]. This could be due to several factors. For instance, a higher abundance of mRNA target genes generally leads to a weaker miRNA activity [45, 46].

Overall, our *in vitro* data suggest that in human cancer cells: 1) 1B3 overexpression leads to an anti-cancer effect, although the precise downstream mechanism varies between cell lines; 2) the anti-cancer effect of 1B3 is due to simultaneous targeting of multiple target genes, rather than one individual gene; and 3) the target genes responsible for the anti-cancer effect of 1B3 vary between cell lines. Thus, the broad anti-cancer effect of 1B3 lends it to a potentially useful and effective agent for therapeutic intervention in oncology. Cancer is a complex, multigenic, and heterogeneous disease, and a single patient will often carry cells with unique genetic origins. Therefore, a miRNA therapeutic which regulates multiple pathways, or multiple elements within a pathway, has the potential to destroy several different tumor subclones and might be less subject to drug treatment-acquired resistance [11, 12]. In support of this concept, combinations of single target agents are already being used in the clinic to more effectively treat cancer [47, 48].

Despite growing evidence of the therapeutic value of miRNAs, effective *in vivo* delivery remains a challenge. Indeed, most *in vivo* investigations of miR-193a-3p implant stably transduced tumor cell lines, rather than treating established experimental tumors. Our study extends the findings of several groups who have shown decreased tumor growth and metastasis of miR-193a-3p transduced cells *in vivo* [38, 43, 49]. Although these data support the use of miR-193a-3p mimics as an anti-cancer therapy, a clinically relevant miRNA must be efficiently delivered to an established tumor. We have recently shown that a chemically modified miR-193a-3p mimic, formulated in a unique lipid nanoparticle, is safely and effectively delivered to tumors *in vivo* (manuscript in preparation). In the current study, we extend these findings by demonstrating that INT-1B3 significantly delays tumor growth in both HCC and melanoma mouse models. Interestingly, Seviour et al. [25] used a lipid nanoparticle formulated miR-193a-3p mimic to treat an orthotopic A549 lung tumor mouse model and, in concordance with our results, reported a significant decrease in tumor growth and metastasis in treated animals.

In summary, we have shown that the miR-193a-3p mimic, 1B3, consistently suppresses several pro-tumorigenic phenotypes *in vitro*. Some of these effects (e.g., viability) can be broadly applied to all cancer cell lines, while others (e.g., cell cycle arrest patterns) are unique to specific cell lines and genetic backgrounds. Using both individual and combined siRNA(s) we showed that no individual 1B3 target reproduced the miRNA effect which suggests that multiple, simultaneous, gene repression by 1B3 is more effective at reducing tumor cell growth than single gene targeting. These data add to the growing body of evidence that miR-193a-3p has an anti-cancer effect through several mechanisms, and that this overall anti-cancer effect is conserved regardless of tumor origin or genetic background. Finally, in two different *in vivo* models we showed a significant reduction of tumor growth. Together, this evidence supports the further clinical development of INT-1B3 as an anti-cancer therapeutic molecule.

## MATERIALS AND METHODS

### Oligonucleotides

The miR-193a-3p mimic, 1B3, and miRNA control 3A1 (unrelated miRNA; based on Thermo Fisher #4464058) were manufactured by BioSpring GmbH (Frankfurt am Mein, Germany). The nucleotide sequences are as follows (with limited 2′-O-methyl nucleotide modifications on the passenger strand [50]):

1B3 passenger (sense) strand: *(3*′*)-*TTUUGACCGGAUGUUUCAGGGU-*(5*′*)*

1B3 guide (anti-sense) strand: *(5*′*)-*AACUGGCCUACAAAGUCCCAGU-*(3*′*)*

3A1 passenger (sense) strand: *(3*′*)-*TTAAUGCAGCAGCGCAGCAAU-*(5*′*)*

3A1 guide (anti-sense) strand: *(5*′*)-*UUACGUCGUCGCGUCGUUATT-*(3*′*)*

Small double stranded oligonucleotides, such as siRNAs or miRNAs targeting RNA, are easily degraded by nucleases and have low membrane permeability due to their high molecular weight and negative charges. Hence, an efficient drug delivery system is needed to improve their therapeutic efficacy *in vivo*. INT-1B3 represents 1B3 formulated in lipid nanoparticles [50] was manufactured by Axolabs GmbH (Kulmback, Germany).

In addition, several siRNAs were used in the presented studies: siPOOL (negative siRNA control) and SMARTpool siRNAs (siCCND1, siCDK6, siKRAS, siMCL1, siSTMN1 and siYWHAZ; which represent siRNAs designed to downregulate cyclin D1, cyclin-dependent kinase 6, K-Ras, myeloid leukemia cell differentiation 1, stathmin 1 and 14-3-3 protein zeta/delta (14-3-3ζ) mRNA, respectively) were obtained from Dharmacon. Each SMARTpool contains four unique siRNA sequences targeting the same mRNA target.

### Cell culture and transfection

The human cell lines Hep3b (HCC), SNU-449 (HCC), H1299 (NSCLC), H1975 (NSCLC), H460 (NSCLC), A2058 (melanoma), BT-549 (TNBC), HCT116 (colon), PANC-1 (pancreatic), and A549 (NSCLC) cells were obtained from ATCC. Human HuH-7 (HCC) cells were obtained from the Japanese Cancer Resources Bank (JCRB). All cell lines were processed and cultured according to the vendor’s instructions. Cells were seeded at 80% confluency into 6-well culture plates 4 h before transient transfection. For single agent transfection the indicated concentrations of either 1B3, 3A1, siPOOL, or SMARTPOOL siRNAs were mixed with Lipofectamine RNAiMax (ThermoFisher) and added dropwise to the cell monolayer. For combination transfection, siRNAs were combined at the concentrations listed in Table 3, mixed with Lipofectamine RNAiMax (Thermofisher), and added dropwise to the cell monolayer. Mock-transfection and untreated conditions were also included in each experiment. After 16 h, transfected cells were dissociated and re-seeded into assay plates. Media was removed and retained, and cells washed with 500 μL TrypL-E before incubating for 5–15 min in TrypLE. Cells were collected in 1 mL fresh media and centrifuged for 5 min at 200x g. Supernatant was aspirated and discarded, and the pellet resuspended in 1 mL fresh media. Using the cell count of the untreated condition cells were re-seeded into the specific assay plates as specified below for each assay.

### Nuclei count and high content cell cycle analysis

For cell count and high content cell cycle analysis, transfected cells were seeded into triplicate wells of black-walled, clear bottom 96-well plates (Greiner) at the appropriate cell density to reach confluence 72 h later. Forty-eight, 72 and 96 h after transfection, media was removed and replaced with 100 μL staining mix containing 0.25% PFA, 0.075% saponin and 1 μg/mL Hoechst 33342 in PBS. Cell imaging was performed on the Thermo CellInsite Automated Imager using the inbuilt high content screening software to identify cell nuclei. Fifteen images/well using the 386_23 filter were captured at 10× magnification. High content cell cycle analysis was performed using nuclei intensity information as described previously [28]. Taxol and siCCND1 were included as positive G2/M and G0/G1 controls, respectively.

### Caspase-Glo 3/7 assay

For Caspase-Glo 3/7 assay, transfected cells were seeded into triplicate wells of white-walled, clear-bottom 96-well plates (Greiner) at the appropriate cell density to reach confluence 72 h later. The Caspase-Glo 3/7 assay (Promega) was performed 48 and 72 h after transfection, according to the manufacturers’ instructions.

### RNA extraction

For RNA extraction, 100–200 thousand transfected cells (dependent on the cell line) were seeded into 12-well tissue culture plates (Greiner). Twenty-four hours after transfection, media was aspirated from the 80% confluent cell monolayers, and frozen at –80°C. RNA was extracted using TRIzol (Thermo Fisher) according to the manufacturers’ instructions. RNA concentration was measured on NanoDrop One (Thermo Fisher).

### Two-tailed RT-qPCR

For the detection of miRNAs *in vitro*, the reverse transcription reaction of 20 ng of total RNAs was performed using 2-tailed RT primers designed by TATAA Biocenter and GrandScript cDNA synthesis Kit (TATAA Biocenter), according to the manufacturer’s instructions. Quantification of the miRNA expression levels were subsequently assessed by quantitative PCR (qPCR) using SYBR Green (Bio-Rad). Results were interpolated with a calibration curve to determine the amount of 1B3 per cell. It is of note that the guide (antisense) strand sequence of 1B3 is identical to the mature human miR-193a-3p. Therefore, primers cannot distinguish between endogenous miR-193a-3p expression and the level of exogenous (transfected) 1B3. All figures are labelled ‘1B3 level’ but it should be assumed that this also includes endogenous miR-193a-3p expression, which is determined in the mock-transfected conditions or PBS control treated animals.

### Stem-loop RT-qPCR

For the detection of miRNAs *in vivo*, the reverse transcription reaction of 100 ng of total RNAs was performed using in-house designed stem-loop RT primers (IDT). Quantification of the miRNA levels were subsequently assessed by quantitative PCR (qPCR) using SYBR Green (Bio-Rad). Cycle threshold (Ct) values were interpolated with a calibration curve to determine the amount of 1B3 per ng tumor tissue. The Stem loop primer sequences used in this study are provided in Supplementary Table 2. As indicated in the previous chapter, the primers used cannot distinguish between endogenous miR-193a-3p expression and the level of exogenous (transfected) 1B3. All figures are labelled ‘1B3 level’ but it should be assumed that this also includes endogenous miR-193a-3p expression, which is determined in the mock-transfected conditions or PBS control-treated animals.

### RT-qPCR

For the detection of target mRNAs, 100 ng of total RNA was first transcribed into single stranded cDNA. One μL synthesized cDNA was used in a 20 μL PCR amplification reaction using SYBR Green master mix (BioRAD) on CFX96 Real-Time qPCR machine according to the manufacturers’ instructions. Triplicate reactions were performed for each condition. The qPCR primer sequences used in this study are listed in Supplementary Table 3. Expression values were calculated using 2^−ΔCt^ method. ΔCt was calculated by subtracting the geometric mean of the Ct of two reference genes from the Ct of the target genes. Values were normalized to mock to determine target downregulation.

### Protein isolation and western blotting

For protein extraction, cells were seeded into 6-well tissue culture plates at the appropriate cell density to reach confluence 72 h later. Cell pellets were collected 24, 48 and 72 h after transfection by trypsinization. One hundred to 200 μL RIPA buffer (50 mM Tris-HCl pH 8, 150 mM NaCl, 1% NP40, 0.5% sodium deoxycholate, 0.1% SDS, 0.5 mM EDTA), supplemented with protease and phosphatase inhibitor cocktails, was added to harvested cells. Lysates were centrifugated at 15,000× g for 1 h at 4°C, and clarified by removing the cell debris pellet. Protein concentration was determined using the Pierce BCA Protein Assay Kit (Thermo Fisher). Samples were separated at 120 V by polyacrylamide gel electrophoresis under denaturing conditions (SDS-PAGE) on Mini-PROTEAN TGX Stain-Free precast gels (Bio-Rad). Proteins were transferred at 200 mA for 2 h to polyvinylidene difluoride (PVDF) membranes in transfer buffer (25 mM Tris, 192 mM glycine, 20% methanol, pH 8.3). The membranes were blocked using 5% milk or 5% BSA in Tris-buffered saline with Tween (20 mM Tris pH 7.6, 137 mM NaCl, 0.1% Tween) to prevent non-specific antibody binding. Blots were probed with primary and horseradish peroxidase-conjugated secondary antibodies. Proteins were detected using enhanced chemiluminescence (ECL) reagents. Membranes were stripped by incubation in stripping buffer (62.5 mM Tris pH 6.8, 2% SDS, 100 mM 2-mercaptoethanol) for 30 min at 50°C and re-probed as appropriate. Band intensities were quantified by densitometry using ImageJ software and values normalized to the tubulin loading control and shown relative to the mock transfected cells.

### SA-β-gal senescence assay

For the SA-β-gal assay, transfected cells were seeded in 6-well cell culture plates (Greiner) at the appropriate cell density to reach confluence 72 h later. One well of untreated cells was seeded in low-serum (0.1% FBS) media as a positive control for senescence. The SA-β-gal assay was performed 96 h after transfection using the SA-β-gal assay kit (Cell Signalling Technology) according to the manufacturer’s instructions. Cells were imaged at 10x magnification using a Leica DMIL LED microscope. A total of 500 cells were counted from each well using the image analysis software Fiji [51].

### Transwell migration assay

For the migration assay, 16 h after transfection in 6-well plates, media was aspirated and replaced in low serum (0.2% FBS) media. After a further 6 h, cells were dissociated as described above and seeded into duplicate wells of 24-well cell culture migration inserts (Corning) at the appropriate cell density to reach confluence 24 h later. Complete media (10% FBS) was added to the bottom well. The cells were allowed to migrate for 24 h at 37°C in a humidified 5% CO_2_ incubator. Forty-eight hours after transfection, unmigrated cells were removed from the chamber with a cotton swab and the chambers fixed with 70% ethanol then stained in a 1% Crystal Violet (Sigma) solution. Using a Leica DMIL LED microscope, 10 images per field at 20x magnification were obtained. The number of migrated cells was counted using the imaging software Fiji [51]. To control for the effect of 1B3 on cell viability, an adjacent 24-well tissue culture plate (Greiner) without the cell culture migration insert was seeded as above. Forty-eight hours after transfection, cells from each condition were dissociated and counted using the TC-10 counter (Bio-Rad) to determine the effect of 1B3 on cell viability. To calculate the proportion of migrated cells, independent of the effect of 1B3 on viability, a viability correction factor was calculated by dividing the counts from the treated well by the mock treated well. The count of migrated cells was then divided by the viability correction factor.

### Experimental tumor models

Studies with the human HCC Hep3B and human melanoma A2058 experimental tumor models were performed at Crown Bioscience Inc (Taicang, China) and Oncotest/Charles River (Freiburg, Germany), respectively. Study protocols were reviewed and approved by the Institutional Animal Care and Use Committee, and were conducted in accordance with the regulations of the Association for Assessment and Accreditation of Laboratory Animal Care. For the experimental Hep3B tumor model, Hep3B tumor cells were first injected into the flank of Balb/c immune-compromized (Nude) mice to form subcutaneous tumors. Then, 7- to 8-week old female SCID/Beige mice were implanted with a single 2-mm^3^ piece of a subcutaneously grown Hep3B tumor into the left liver lobe (orthotopic tumor model). Tumor development was monitored by measurement of serum alpha-fetoprotein (AFP) which serves as tumor marker and indicator of liver tumor development [31]. When increasing serum AFP values reached a median of 5.2 μg/mL (indicating the presence of established tumors), animals were randomized in the various experimental groups, and were treated with either vehicle (PBS) or 10 mg/kg/administration of INT-1B3 by repeated bolus injections (intravenous; i.v) for 3 consecutive days in the first week, and maintained on a twice weekly (‘Monday/Thursday’) schedule for an additional three weeks. Serum AFP levels were measured weekly throughout the study. For the experimental A2058 tumor model, 6- to 8-week old female BALB/c Nude mice were subcutaneously injected in the right flank with 5 × 10^6^ A2058 cells in 0.1-mL PBS. Once the mean subcutaneous tumor size reached ~150 mm^3^ (i.e., established subcutaneous tumors), animals were randomized in the various experimental groups, and animals were treated with either vehicle (PBS) or 3 mg/kg/administration of INT-1B3 by repeated bolus injection (i.v) daily for 5 days during the first week, then maintained on a twice weekly (‘Monday/Thursday’) schedule for the next 2 weeks. Tumor volume was monitored by caliper-aided measurements twice a week throughout the study.

## SUPPLEMENTARY MATERIALS



## Abbreviations

AFP: alpha fetoprotein
B2M: Beta-2 microglobulin
*CCND1*: Cyclin D1
*CCND2*: Cyclin D2
*CCNE2*: Cyclin E2
*CDK2*: Cyclin dependent kinase 2
*CDK4*: Cyclin dependent kinase 4
*CDK6*: Cyclin dependent kinase 6
*cKIT*: Proto-oncogene c-KIT
Ct: Cycle threshold
EDTA: Ethylenediaminetetraacetic acid
FBS: Fetal bovine serum
ɣH2AX: phosphorylated histone family member X
*GRB7*: Growth factor receptor-bound protein 7
HCC: hepatocellular carcinoma
*HMGB1*: High mobility group box 1
i.v: intravenous
*KRAS*: KRAS Proto-Oncogene
*MCL1*: myeloid leukemia cell differentiation protein
NaCl: Sodium chloride
*PAK4*: Serine/threonine-protein kinase PAK 4
PARP: poly (ADP-ribose) polymerase
PBS: phosphate buffered saline
PFA: paraformaldehyde
PI: propidium iodide
*PLAU*: plasminogen activator, urokinase
PTEN: phosphatase and tensin homolog
*PTP1B*: protein-tyrosine phosphatase 1B
*RAB7A*: RAB7A, member RAS oncogene family
*RASSF1*: Ras association domain family member 1
ROS: reactive oxygen species
*S6K2*: 40S ribosomal S6 kinase 2
SA-β-gal: Senescence associated beta-galactosidase
SDS: Sodium dodecyl sulfate
*STMN1*: Stathmin 1
T/C: tumor/control
*TP53*: Tumor protein p53
*UBC*: Ubiquitin C
V: volts
x g: relative centrifugal force

## References

[1] Ferlay J , Colombet M , Soerjomataram I , Mathers C , Parkin DM , Piñeros M , Znaor A , Bray F . Estimating the global cancer incidence and mortality in 2018: GLOBOCAN sources and methods. Int J Cancer. 2019; 144:1941–53. 10.1002/ijc.31937. 30350310

[2] Hanahan D , Weinberg RA . Hallmarks of cancer: The next generation. Cell. 2011; 144:646–74. 10.1016/j.cell.2011.02.013. 21376230

[3] Hausser J , Alon U . Tumour heterogeneity and the evolutionary trade-offs of cancer. Nat Rev Cancer. 2020; 20:247–57. 10.1038/s41568-020-0241-6. 32094544

[4] Pasquinelli AE . MicroRNAs and their targets: recognition, regulation and an emerging reciprocal relationship. Nat Rev Genet. 2012; 13:271–82. 10.1038/nrg3162. 22411466

[5] Hausser J , Zavolan M . Identification and consequences of miRNA-target interactions-beyond repression of gene expression. Nat Rev Genet. 2014; 15:599–612. 10.1038/nrg3765. 25022902

[6] Vidigal JA , Ventura A . The biological functions of miRNAs: lessons from *in vivo* studies. Trends Cell Biol. 2015; 25:137–47. 10.1016/j.tcb.2014.11.004. 25484347 PMC4344861

[7] Croce CM . Causes and consequences of microRNA dysregulation in cancer. Nat Rev Genet. 2009; 10:704–14. 10.1038/nrg2634. 19763153 PMC3467096

[8] Peng Y , Croce CM . The role of MicroRNAs in human cancer. Signal Transduct Target Ther. 2016; 1:15004. 10.1038/sigtrans.2015.4. 29263891 PMC5661652

[9] Chakraborty C , Sharma AR , Sharma G , Doss CGP , Lee SS . Therapeutic miRNA and siRNA: Moving from Bench to Clinic as Next Generation Medicine. Mol Ther Nucleic Acids. 2017; 8:132–43. 10.1016/j.omtn.2017.06.005. 28918016 PMC5496203

[10] Rupaimoole R , Slack FJ . MicroRNA therapeutics: towards a new era for the management of cancer and other diseases. Nat Rev Drug Discov. 2017; 16:203–22. 10.1038/nrd.2016.246. 28209991

[11] Hanna J , Hossain GS , Kocerha J . The Potential for microRNA Therapeutics and Clinical Research. Front Genet. 2019; 10:478. 10.3389/fgene.2019.00478. 31156715 PMC6532434

[12] To KKW , Fong W , Tong CWS , Wu M , Yan W , Cho WCS . Advances in the discovery of microRNA-based anticancer therapeutics: latest tools and developments. Expert Opin Drug Discov. 2019; 15:63–83. 10.1080/17460441.2020.1690449. 31739699

[13] Bonneau E , Neveu B , Kostantin E , Tsongalis GJ , De Guire V . How close are miRNAs from clinical practice? A perspective on the diagnostic and therapeutic market. EJIFCC. 2019; 30:114–27. 31263388 PMC6599191

[14] Poell JB , van Haastert RJ , de Gunst T , Schultz IJ , Gommans WM , Verheul M , Cerisoli F , van Noort PI , Prevost GP , Schaapveld RQJ , Cuppen E . A Functional Screen Identifies Specific MicroRNAs Capable of Inhibiting Human Melanoma Cell Viability. PLoS One. 2012; 7:e43569. 10.1371/journal.pone.0043569. 22927992 PMC3425484

[15] Grossi I , Salvi A , Abeni E , Marchina E , De Petro G . Biological Function of MicroRNA193a-3p in Health and Disease. Int J Genomics. 2017; 2017:5913195. 10.1155/2017/5913195. 29038785 PMC5605928

[16] Yu M , Liu Z , Liu Y , Zhou X , Sun F , Liu Y , Li L , Hua S , Zhao Y , Gao H , Zhu Z , Na M , Zhang Q , et al. PTP1B markedly promotes breast cancer progression and is regulated by miR-193a-3p. FEBS J. 2019; 286:1136–53. 10.1111/febs.14724. 30548198

[17] Mamoori A , Wahab R , Islam F , Lee K , Vider J , Lu CT , Gopalan V , Lam AK . Clinical and biological significance of miR-193a-3p targeted KRAS in colorectal cancer pathogenesis. Hum Pathol. 2018; 71:145–56. 10.1016/j.humpath.2017.10.024. 29104111

[18] Wang J , Yang B , Han L , Li X , Tao H , Zhang S , Hu Y . Demethylation of miR-9-3 and miR-193a genes suppresses proliferation and promotes apoptosis in non-small cell lung cancer cell lines. Cell Physiol Biochem. 2013; 32:1707–19. 10.1159/000356605. 24356455

[19] Kwon JE , Kim BY , Kwak SY , Bae IH , Han YH . Ionizing radiation-inducible microRNA miR-193a-3p induces apoptosis by directly targeting Mcl-1. Apoptosis. 2013; 18:896–909. 10.1007/s10495-013-0841-7. 23546867

[20] Fan Q , Hu X , Zhang H , Wang S , Zhang H , You C , Zhang CY , Liang H , Chen X , Ba Y . MiR-193a-3p is an Important Tumour Suppressor in Lung Cancer and Directly Targets KRAS. Cell Physiol Biochem. 2017; 44:1311–24. 10.1159/000488131. 29183007

[21] Pruikkonen S , Kallio MJ . Excess of a Rassf1-targeting microRNA, miR-193a-3p, perturbs cell division fidelity. Br J Cancer. 2017; 116:1451–61. 10.1038/bjc.2017.110. 28449010 PMC5520089

[22] Lin M , Zhang Z , Gao M , Yu H , Sheng H , Huang J . MicroRNA-193a-3p suppresses the colorectal cancer cell proliferation and progression through downregulating the PLAU expression. Cancer Manag Res. 2019; 11:5353–63. 10.2147/cmar.s208233. 31354344 PMC6578599

[23] Hydbring P , Wang Y , Fassl A , Li X , Matia V , Otto T , Choi YJ , Sweeney KE , Suski JM , Yin H , Bogorad RL , Goel S , Yuzugullu H , et al. Cell-Cycle-Targeting MicroRNAs as Therapeutic Tools against Refractory Cancers. Cancer Cell. 2017; 31:576–590.e8. 10.1016/j.ccell.2017.03.004. 28399412 PMC5425285

[24] Ma P , Wang H , Sun J , Liu H , Zheng C , Zhou X , Lu Z . LINC00152 promotes cell cycle progression in hepatocellular carcinoma via miR-193a/b-3p/CCND1 axis. Cell Cycle. 2018; 17:974–84. 10.1080/15384101.2018.1464834. 29895195 PMC6103663

[25] Seviour E , Sehgal V , Mishra D , Rupaimoole R , Rodriguez-Aguayo C , Lopez-Berestein G , Lee JS , Sood A , Kim M , Mills G , Ram P . Targeting KRas-dependent tumour growth, circulating tumour cells and metastasis *in vivo* by clinically significant miR-193a-3p. Oncogene. 2017; 36:1339–50. 10.1038/onc.2016.308. 27669434 PMC5344721

[26] Bharambe HS , Joshi A , Yogi K , Kazi S , Shirsat NV . Restoration of miR-193a expression is tumor-suppressive in MYC amplified Group 3 medulloblastoma. Acta Neuropathol Commun. 2020; 8:70. 10.1186/s40478-020-00942-5. 32410663 PMC7227220

[27] van den Bosch MTJ , Yahyanejad S , Alemdehy MF , Telford BJ , de Gunst T , den Boer HC , Vos RM , Stegink M , van Pinxteren LAH , Schaapveld RQJ , Janicot M . Transcriptome- wide analysis reveals insight into tumor suppressor functions of 1B3, a novel synthetic miR- 193a- 3p mimic. Mol Ther Nucleic Acids. 2021. [Epub ahead of print]. 10.1016/j.omtn.2021.01.020. PMC7896128 33664995

[28] Chan GKY , Kleinheinz TL , Peterson D , Moffat JG . A Simple High-Content Cell Cycle Assay Reveals Frequent Discrepancies between Cell Number and ATP and MTS Proliferation Assays. PLoS One. 2013; 8:e63583. 10.1371/journal.pone.0063583. 23691072 PMC3656927

[29] Hernandez-Segura A , Nehme J , Demaria M . Hallmarks of Cellular Senescence. Trends Cell Biol. 2018; 28:436–53. 10.1016/j.tcb.2018.02.001. 29477613

[30] Rayburn ER , Zhang R . Antisense, RNAi, and gene silencing strategies for therapy: mission possible or impossible? Drug Discov Today. 2008; 13:513–21. 10.1016/j.drudis.2008.03.014. 18549978 PMC2497463

[31] Zhang J , Chen G , Zhang P , Zhang J , Li X , Gan D , Cao X , Han M , Du H , Ye Y . The threshold of alpha-fetoprotein (AFP) for the diagnosis of hepatocellular carcinoma: A systematic review and meta-analysis. PLoS One. 2020; 15:e0228857. 10.1371/journal.pone.0228857. 32053643 PMC7018038

[32] Liu Y , Ren F , Luo Y , Rong M , Chen G , Dang Y . Down-Regulation of MiR-193a-3p Dictates Deterioration of HCC: A Clinical Real-Time qRT-PCR Study. Med Sci Monit. 2015; 21:2352–60. 10.12659/msm.894077. 26263159 PMC4538786

[33] Salvi A , Conde I , Abeni E , Arici B , Grossi I , Specchia C , Portolani N , Barlati S , De Petro G . Effects of miR-193a and sorafenib on hepatocellular carcinoma cells. Mol Cancer. 2013; 12:162. 10.1186/1476-4598-12-162. 24330766 PMC4029516

[34] Liu Y , Xu X , Xu X , Li S , Liang Z , Hu Z , Wu J , Zhu Y , Jin X , Wang X , Lin Y , Chen H , Mao Y , et al. MicroRNA-193a-3p inhibits cell proliferation in prostate cancer by targeting cyclin D1. Oncol Lett. 2017; 14:5121–8. 10.3892/ol.2017.6865. 29142597 PMC5666665

[35] Cox AD , Fesik SW , Kimmelman AC , Luo J , Der CJ . Drugging the undruggable Ras: mission possible? Nat Rev Drug Discov. 2014; 13:828–51. 10.1038/nrd4389. 25323927 PMC4355017

[36] Waters AM , Der CJ . KRAS: The Critical Driver and Therapeutic Target for Pancreatic Cancer. Cold Spring Harb Perspect Med. 2018; 8:a031435. 10.1101/cshperspect.a031435. 29229669 PMC5995645

[37] Bartel DP . MicroRNAs: target recognition and regulatory functions. Cell. 2009; 162:215–33. 10.1016/j.cell.2009.01.002. 19167326 PMC3794896

[38] Yu T , Li J , Yan M , Liu L , Lin H , Zhao F , Sun L , Zhang Y , Cui Y , Zhang F , He X , Yao M . MicroRNA-193a-3p and -5p suppress the metastasis of human non-small-cell lung cancer by downregulating the ERBB4/PIK3R3/mTOR/S6K2 signaling pathway. Oncogene. 2015; 34:413–23. 10.1038/onc.2013.574. 24469061

[39] Gao XN , Lin J , Li YH , Gao L , Wang XR , Wang W , Kang HY , Yan GT , Wang LL , Yu L . MicroRNA-193a represses c-kit expression and functions as a methylation-silenced tumor suppressor in acute myeloid leukemia. Oncogene. 2011; 30:3416–28. 10.1038/onc.2011.62. 21399664

[40] Wu H , Zhou C . Long non-coding RNA UCA1 promotes lung cancer cell proliferation and migration via microRNA-193a/HMGB1 axis. Biochem Biophys Res Commun. 2018; 496:738–45. 10.1016/j.bbrc.2018.01.097. 29355524

[41] Tang Y , Yang S , Wang M , Liu D , Liu Y , Zhang Y , Zhang Q . Epigenetically altered miR-193a-3p promotes HER2 positive breast cancer aggressiveness by targeting GRB7. Int J Mol Med. 2019; 43:2352–60. 10.3892/ijmm.2019.4167. 31017268 PMC6488183

[42] Iliopoulos D , Rotem A , Struhl K . Inhibition of miR-193a Expression by Max and RXRα Activates K-Ras and PLAU to Mediate Distinct Aspects of Cellular Transformation. Cancer Res. 2011; 71:5144–53. 10.1158/0008-5472.CAN-11-0425. 21670079 PMC3148313

[43] Liu X , Min S , Wu N , Liu H , Wang T , Li W , Shen Y , Zhao C , Wang H , Qian Z , Xu H , Chen Y , Wang X . miR-193a-3p Inhibition of the Slug Activator PAK4 Suppresses Non-Small Cell Lung Cancer Aggressiveness via the p53/Slug/L1CAM Pathway. Cancer Lett. 2019; 447:56–65. 10.1016/j.canlet.2019.01.027. 30685413

[44] Clark PM , Loher P , Quann K , Brody J , Londin ER , Rigoutsos I . Argonaute CLIP-Seq reveals miRNA targetome diversity across tissue types. Sci Rep. 2014; 4:5947. 10.1038/srep05947. 25103560 PMC4894423

[45] Mullokandov G , Baccarini A , Ruzo A , Jayaprakash AD , Tung N , Israelow B , Evans MJ , Sachidanandam R , Brown BD . High-throughput assessment of microRNA activity and function using microRNA sensor and decoy libraries. Nat Methods. 2012; 9:840–6. 10.1038/nmeth.2078. 22751203 PMC3518396

[46] Plotnikova O , Baranova A , Skoblov M . Comprehensive Analysis of Human microRNA–mRNA Interactome. Front Genet. 2019; 10:933. 10.3389/fgene.2019.00933. 31649721 PMC6792129

[47] Yap TA , Omlin A , De Bono JS . Development of Therapeutic Combinations Targeting Major Cancer Signaling Pathways. J Clin Oncol. 2013; 31:1592–605. 10.1200/JCO.2011.37.6418. 23509311

[48] Mokhtari RB , Homayouni TS , Baluch N , Morgatskaya E , Kumar S , Das B , Yeger H . Combination therapy in combating cancer. Oncotarget. 2017; 8:38022–43. 10.18632/oncotarget.16723. 28410237 PMC5514969

[49] Liang H , Liu M , Yan X , Zhou Y , Wang W , Wang X , Fu Z , Wang N , Zhang S , Wang Y , Zen K , Zhang CY , Hou D , et al. MiR-193a-3p Functions as a Tumor Suppressor in Lung Cancer by Down-regulating ERBB4. J Biol Chem. 2015; 290:926–40. 10.1074/jbc.M114.621409. 25391651 PMC4294520

[50] De Gunst MM , van Pinxteren LAH , Janicot M , Schultz IJ , Schaapveld RQJ , Yahyanehad S . Interna Technologies B.V. [NL]. Anticancer microRNA and lipid formulations therof. WO/2019/155094. 2019.

[51] Schindelin J , Arganda-Carreras I , Frise E , Kaynig V , Longair M , Pietzsch T , Preibisch S , Rueden C , Saalfeld S , Schmid B , Tinevez JY , White DJ , Hartenstein V , et al. Fiji: An open-source platform for biological-image analysis. Nat Methods. 2012; 9:676–82. 10.1038/nmeth.2019. 22743772 PMC3855844

