# Supplementary material for: Multi-modal effects of 1B3, a novel synthetic miR-193a-3p mimic, support strong potential for therapeutic intervention in oncology
Source: Oncotarget. 2021 Mar 2;12(5):422–39. doi: 10.18632/oncotarget.27894 (PMC7939530; doi:10.18632/oncotarget.27894)
Supplement: Supplementary file 1 [file oncotarget-12-422-s001.pdf]

Multi-modal effects of 1B3, a novel synthetic miR-193a-3p mimic, support strong potential for therapeutic intervention in oncology

SUPPLEMENTARY MATERIALS

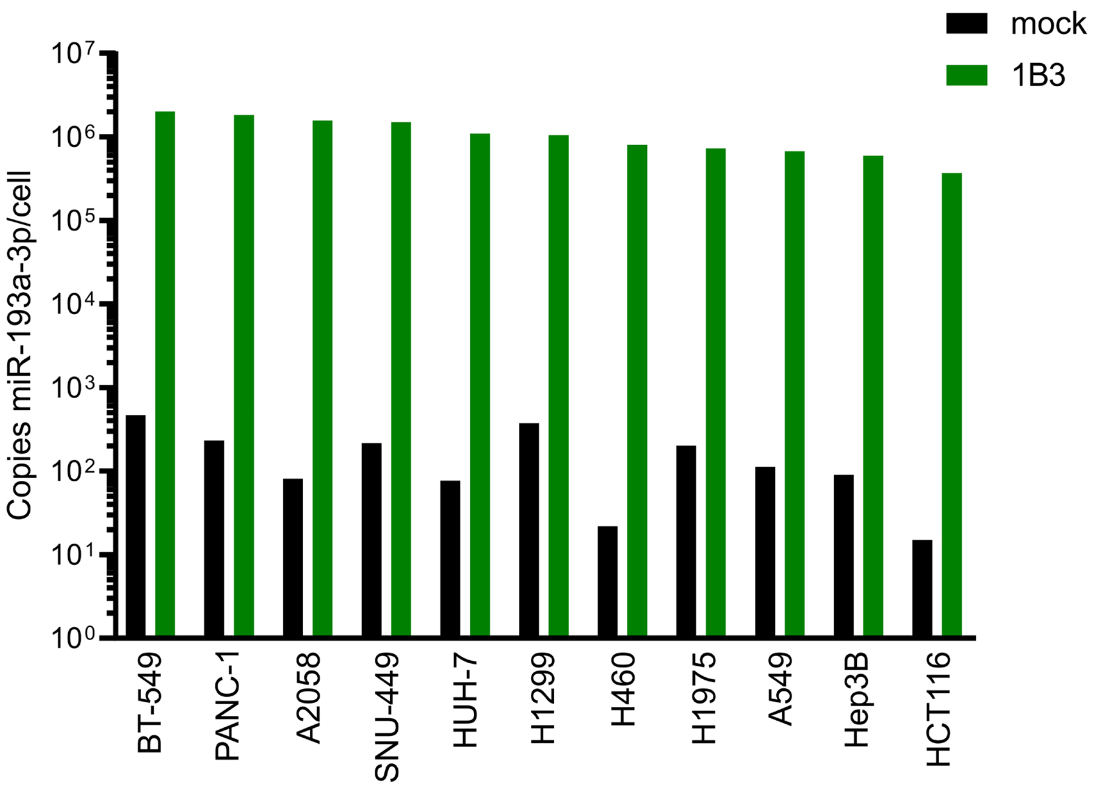

Supplementary Figure 1: Effect of 1B3 transfection on 1B3 levels in a range of human cancer cell lines. Cells were transfected with 10-nM 1B3 in the presence of RNAiMAX transfection reagent. Non-transfected cells (mock) were also included to define endogenous miR-193a-3p levels. RNA was harvested from confluent cell monolayers after 24 h and extracted using TRIzol. 2-tailed RT-qPCR to detect miR-193a-3p/1B3 was performed using 20 ng total RNA. Amount of 1B3/miR-193a-3p was calculated by interpolating to a standard curve to determine copy number then normalizing to the number of cells. As indicated in the Materials and Methods section, the guide (antisense) strand sequence of 1B3 is identical to the mature human miR-193a-3p, and the primers therefore cannot distinguish between endogenous miR-193a-3p expression and the level of exogenous (transfected) 1B3. The y-axis is labelled 'copies miR-193a-3p/cell' but it should be assumed that this also includes 1B3 in the transfected conditions. Bars show the mean of triplicate reactions from one biological replicate.

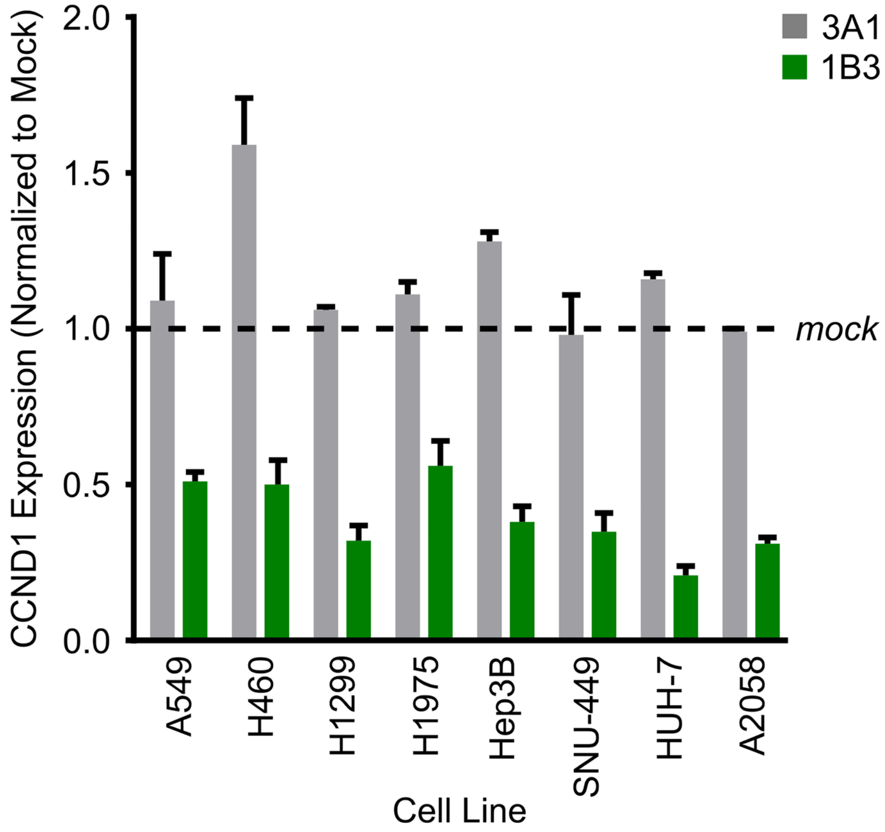

Supplementary Figure 2: Effect of 1B3 transfection on CCND1 mRNA expression in a range of human cancer cell lines. Cells were transfected with 10-nM of either 1B3 or 'negative' miRNA control (3A1) in the presence of RNAiMAX transfection reagent. Non-transfected cells (mock) were also included to define baseline readout. RNA was harvested from confluent cell monolayers after 24 h and extracted using TRIzol. RT-qPCR was used to detect cyclin D1 (*CCND1*) mRNA level, and expression calculated relative to the geometric mean of two reference genes (*B2M* and *UBC*) using $2^{-\Delta CT}$ method. Values were normalized to mock. Error bars show the standard deviation of three independent replicates.

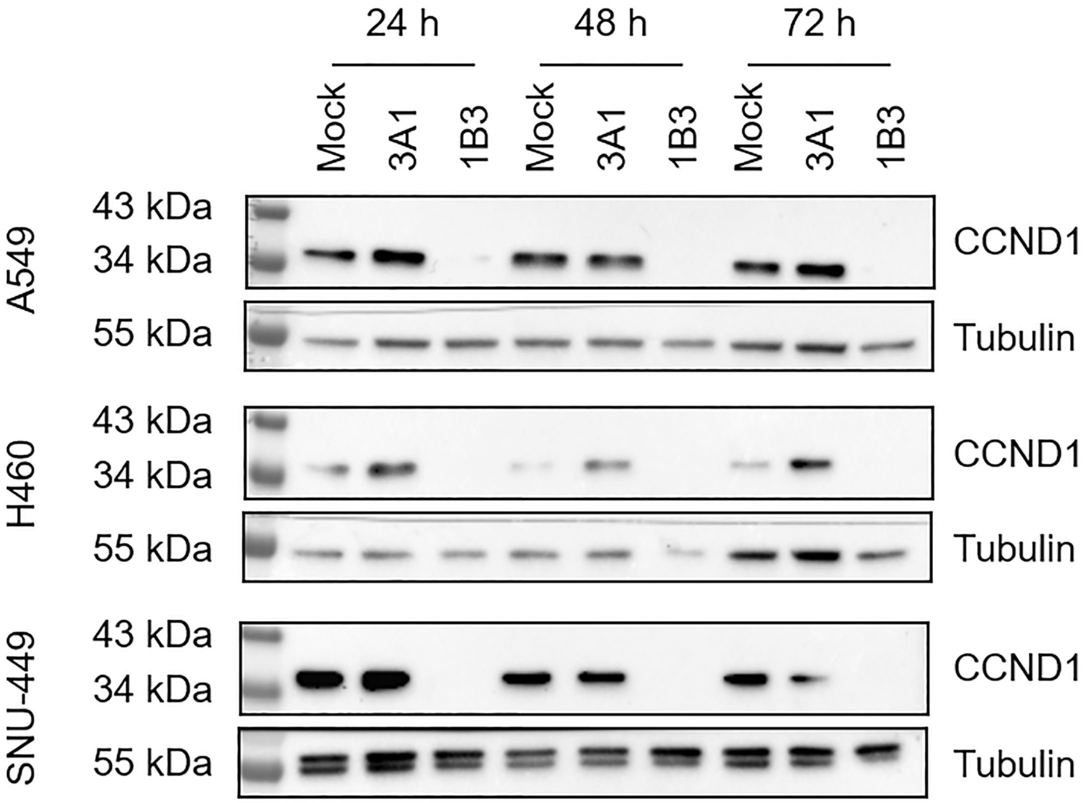

Supplementary Figure 3: Effect of 1B3 transfection on CCND1 protein expression in a range of human cancer cell lines. Cells were transfected with 10-nM of either 1B3 or 'negative' miRNA control (3A1) in the presence of RNAiMAX transfection reagent. Non-transfected cells (mock) were also included to define baseline readout. Cell lysates for Western blotting were harvested 24, 48, and 72 h after transfection. Ten to 20 µg protein was separated by SDS-PAGE, transferred to PVDF membranes, and hybridized with anti-cyclin D1 (CCND1) or tubulin antibodies. Tubulin was used as a protein loading control.

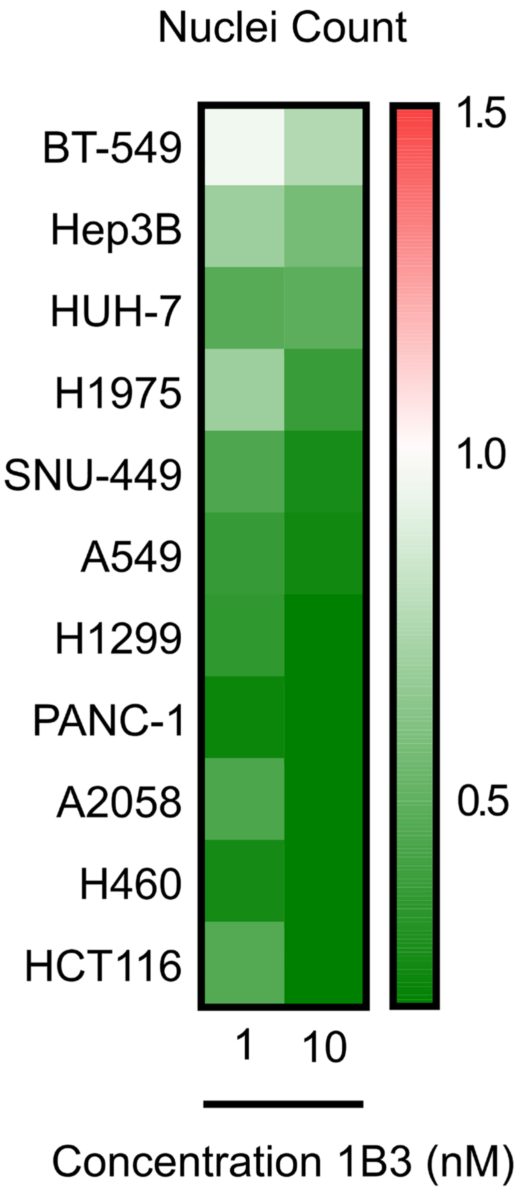

Supplementary Figure 4: Effect of 1B3 transfection on nuclei count in a range of human cancer cell lines. Cells were transfected with either 1B3 or 'negative' miRNA control (3A1) in the presence of RNAiMAX transfection reagent. Non-transfected cells (mock) were also included to define baseline readout. Nuclei count was determined 96 h after transfection with the indicated concentration of 1B3 by staining with Hoeschst-33342 and imaging using the Thermo CellInsite Automated Imager. Values were normalized to mock transfected cells. All values are the mean of at least three independent replicates.

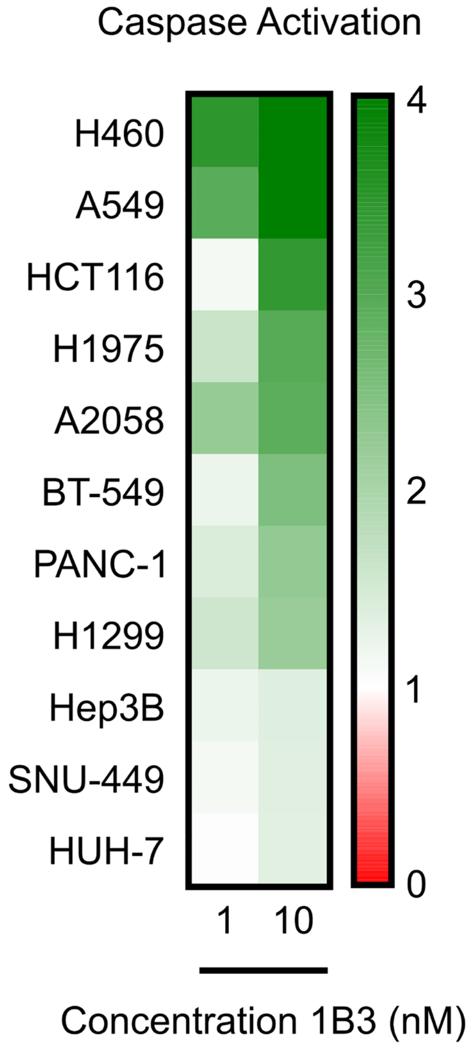

Supplementary Figure 5: Effect of 1B3 transfection on apoptosis in a range of human cancer cell lines. Cells were transfected with either 1B3 or 'negative' miRNA control (3A1) in the presence of RNAiMAX transfection reagent. Non-transfected cells (mock) were also included to define baseline readout. Caspase activation was measured at 48 or 72 h after transfection with the indicated concentration of 1B3 using Caspase-Glo 3/7 assay (Promega). Values were normalized to mock transfected cells. The time point with the highest caspase activation is shown. All values are the mean of at least three independent replicates.

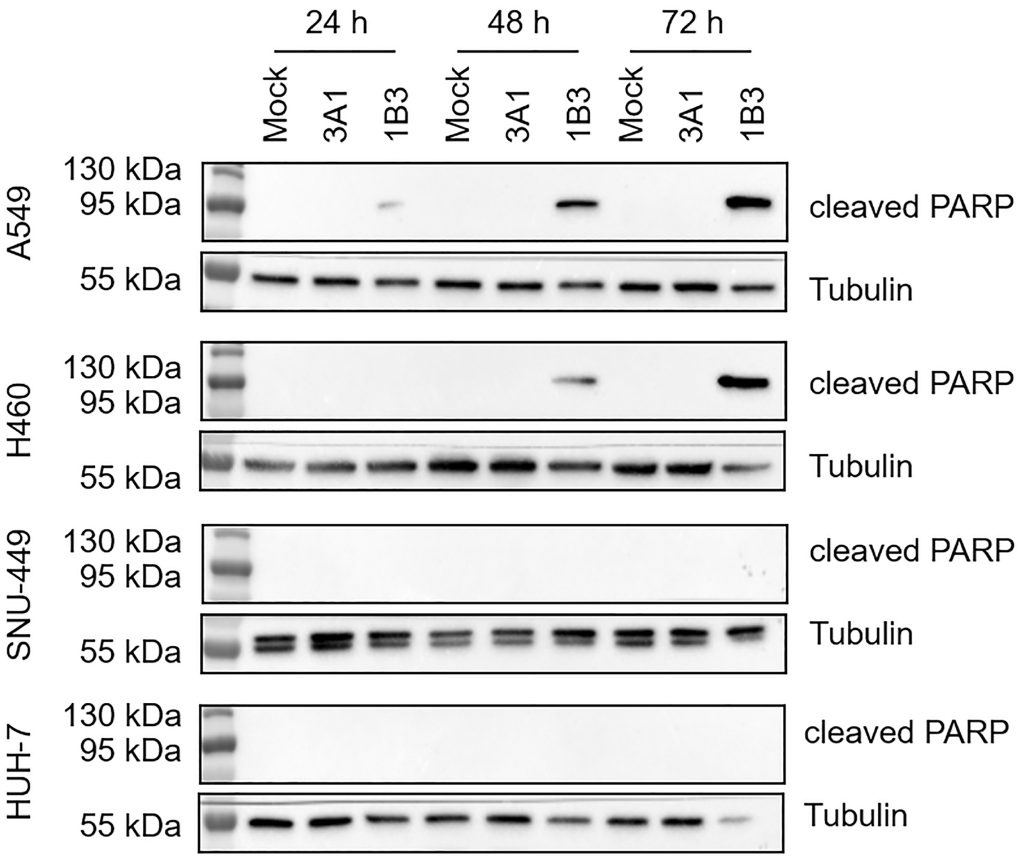

Supplementary Figure 6: Effect of 1B3 transfection on cleaved PARP in a range of human cancer cell lines. Cells were transfected with 10-nM of either 1B3 or 'negative' miRNA control (3A1) in the presence of RNAiMAX transfection reagent. Non-transfected cells (mock) were also included to define baseline readout. Cell lysates for Western blotting were harvested 24, 48, and 72 h after transfection. Ten to 20 µg protein was separated by SDS-PAGE, transferred to PVDF membranes, and hybridized with anti-cleaved *poly (ADP-ribose) polymerase* (*PARP*) or tubulin antibodies. Tubulin was used as a protein loading control.

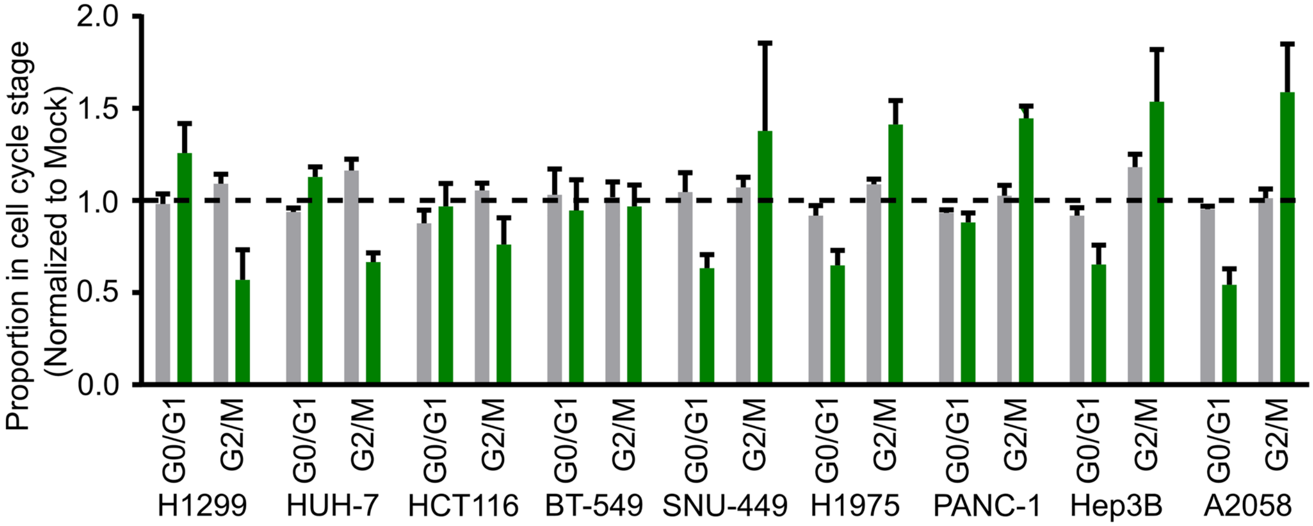

Supplementary Figure 7: Effect of 1B3 transfection on cell cycle in a range of human cancer cell lines. Cells were transfected with either 1B3 or 'negative' miRNA control (3A1) in the presence of RNAiMAX transfection reagent. Non-transfected cells (mock) were also included to define baseline readout. Cell cycle was assayed at 48, 72 or 96 h after transfection with 10-nM of either 3A1 or 1B3, and image-based DNA content analysis used to determine the proportion in each phase of the cell cycle. Values were normalized to mock transfected cells and the optimal time point shown. The dashed line represents the mock value of 1. All values are the mean of at least three independent replicates. Error bars show standard deviation.

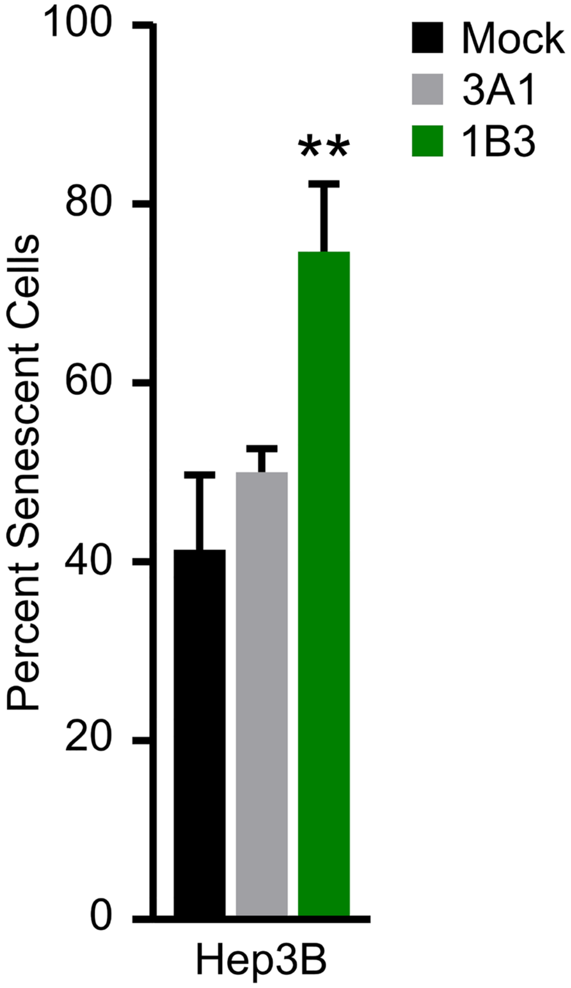

Supplementary Figure 8: Effect of 1B3 transfection on senescence in human HCC Hep3B cells. Cells were transfected with 10-nM of either 1B3 or 'negative' miRNA control (3A1) in the presence of RNAiMAX transfection reagent. Non-transfected cells (mock) were also included to define baseline readout. Senescence was assayed after four days using the SA-β-galactosidase assay (Cell Signaling), and 500 cells in total counted for each condition. The proportion of senescent cells relative to non-senescent cells was calculated. Error bars show the standard deviation of three biological replicates. ** indicates $p < 0.01$ calculated by Dunnett's multiple comparison test.

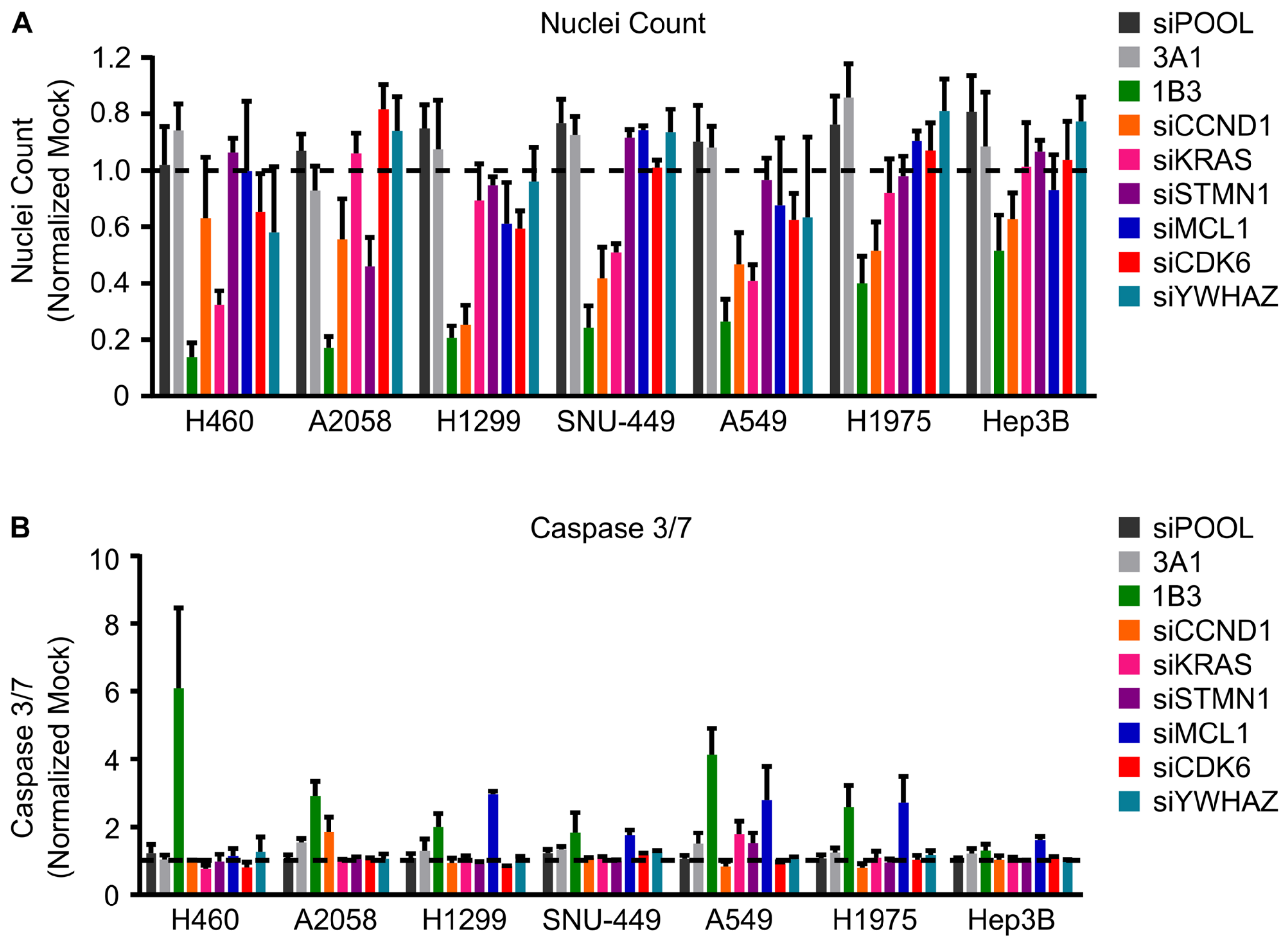

Supplementary Figure 9: Effect of silencing individual 1B3 target gene expression on cell proliferation and apoptosis, in a panel of human cancer cell lines. Cells were transfected with 10-nM of either 1B3, 'negative' miRNA control (3A1), negative control siRNA (siPOOL), or individual siRNAs in the presence of RNAiMAX transfection reagent. Non-transfected cells (mock) were also included to define baseline readout. (**A**) Nuclei count was determined after 96 h by staining with Hoeschst 33342 and imaging using the Thermo CellInsite Automated Imager. Values were normalized to mock transfected cells and represent the mean of three independent replicates. (**B**) Caspase activation was measured 48 or 72 h after transfection using Caspase-Glo 3/7 assay (Promega). Values were normalized to mock transfected cells and represent the mean of three independent replicates. The time point with the highest caspase activation is shown. Error bars show the standard deviation of at least three independent replicates. Dashed line represents the mock value of 1.

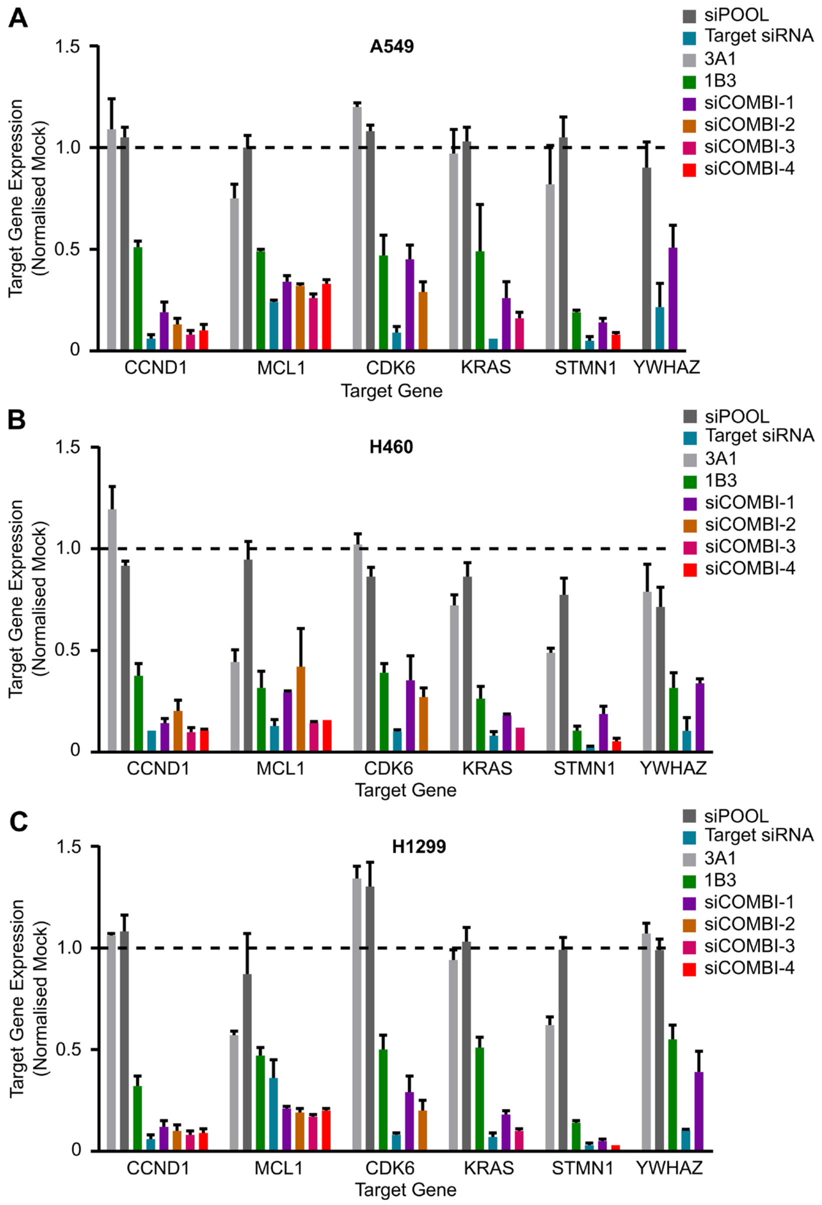

Supplementary Figure 10: Effect of silencing individual, or combinations of 1B3 targets on target gene expression in human cancer cell lines. Human A549 (**A**), H460 (**B**), or H1299 (**C**) cells were transfected in the presence of RNAiMAX transfection reagent with 10-nM of either 1B3, 'negative' miRNA control (3A1), negative control siRNA (siPOOL), or individual siRNAs, or a combination of siRNAs (see Table 2 for compositions) with a total RNAi concentration of 9–14 nM. Non-transfected cells (mock) were also included to define baseline readout. RNA was harvested from confluent cell monolayers after 24 h and extracted using TRIzol. RT-qPCR was used to detect target gene levels and expression calculated relative to the geometric mean of two reference genes (*B2M* and *UBC*) using $2^{-\Delta CT}$ method. Values were normalized to mock (mock = 1). Error bars show the standard deviation of at least two independent replicates. Dashed line represents the mock value of 1.

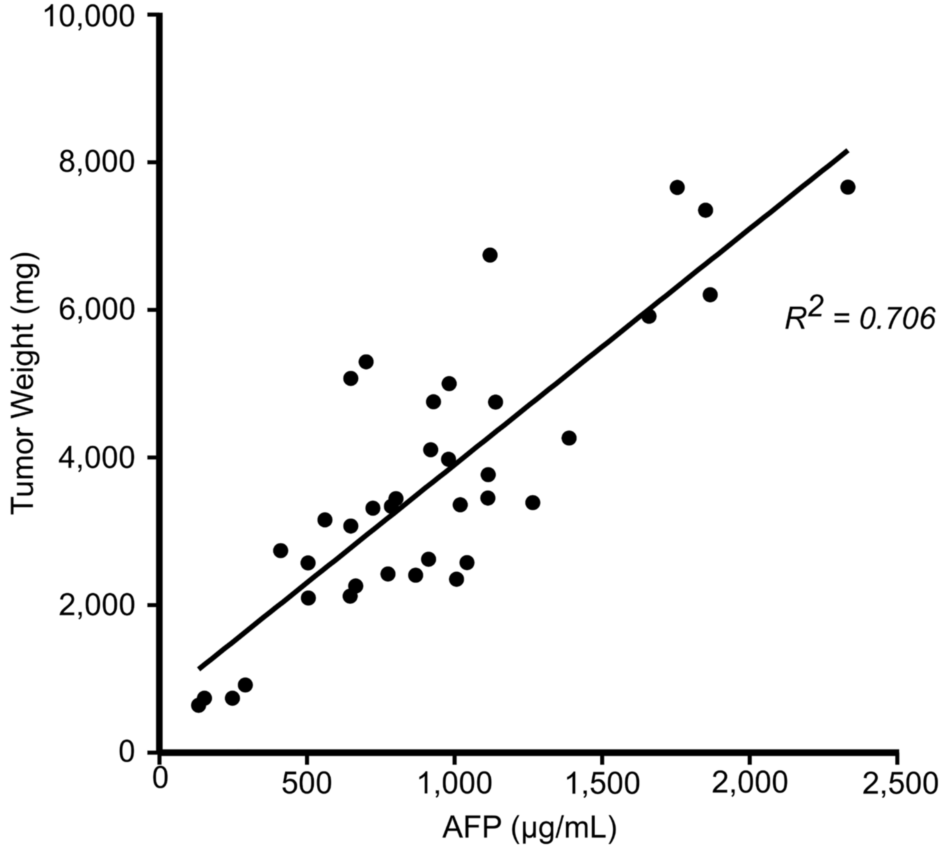

Supplementary Figure 11: Correlation of serum AFP levels with tumor weights on Day 49 in orthotopic human HCC Hep3B tumor-bearing immune-compromised mouse model. Human HCC Hep3B tumor cells were injected in the flank of Balb/c mice to form subcutaneous tumors. Once appropriate tumor size was reached, tumors were collected, and ~2-mm^3 fragments were implanted into the left liver lobe (orthotopic model) of SCID/Beige mice. Serum AFP levels were measured as indicator of liver tumor development. On day 21, animals were randomized in experimental groups based on increasing serum AFP levels (median ~5.0 µg/mL), and treatment with test items started one day after (*i.e.*, on Day 22). On day 49, serum AFP was measured immediately prior to animal sacrifice and tumor collection. The line shows the linear regression between tumor weight and AFP levels (R^2 = 0.706).

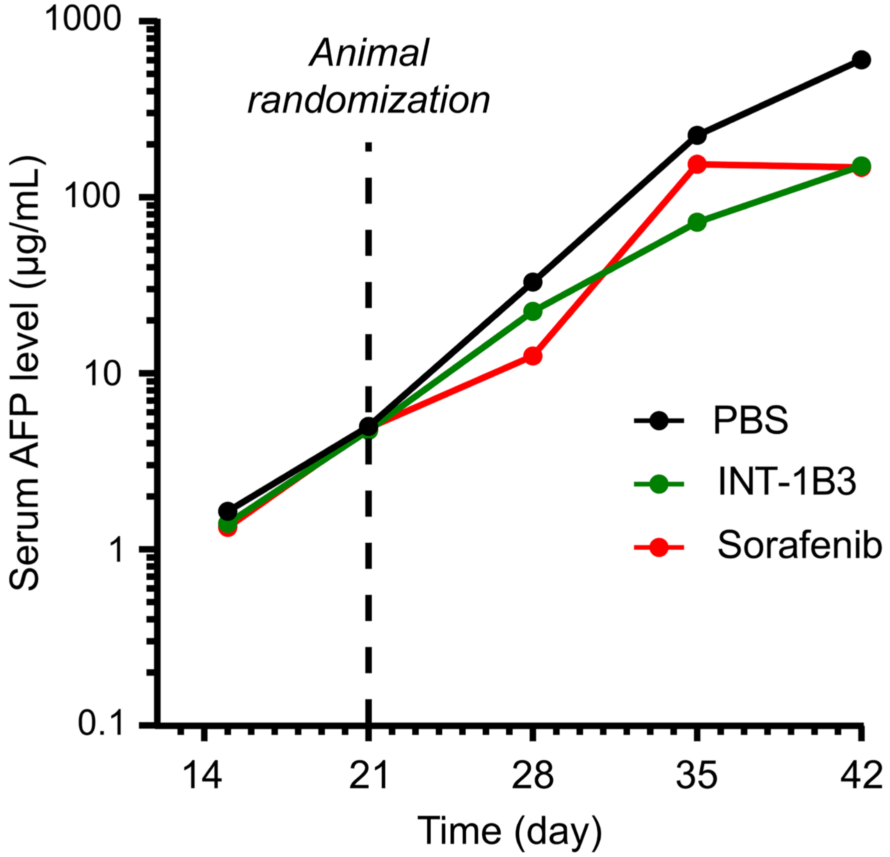

Supplementary Figure 12: Effect of INT-1B3 in orthotopic human HCC Hep3B tumor-bearing immune-compromised mouse model. Human HCC Hep3B tumor cells were injected in the flank of Balb/c mice to form subcutaneous tumors. Once appropriate tumor size was reached, tumors were collected, and ~2-mm^3 fragments were implanted into the left liver lobe (orthotopic model) of SCID/Beige mice. Serum AFP levels were measured as indicator of liver tumor development. On day 21, animals were randomized in experimental groups based on increasing serum AFP levels (median ~5.0 µg/mL), and treatment with test items started one day after (*i.e.*, on Day 22). Animals were treated with either vehicle (PBS, i.p.; Black) or INT-1B3 (10 mg/kg/administration, i.v.; Green) daily for three days during the first week, then twice weekly for the following two weeks. Animals were also treated with sorafenib (10 mg/kg/administration, p.o., BID; Red) as positive control in this experimental tumor model. Serum AFP levels were determined weekly throughout the study. Graph shows the time-dependent evolution of serum AFP levels (median, expressed as µg/mL) represented on a semi-log scale due to the observed ~3 order of magnitude increase in AFP levels throughout the study.

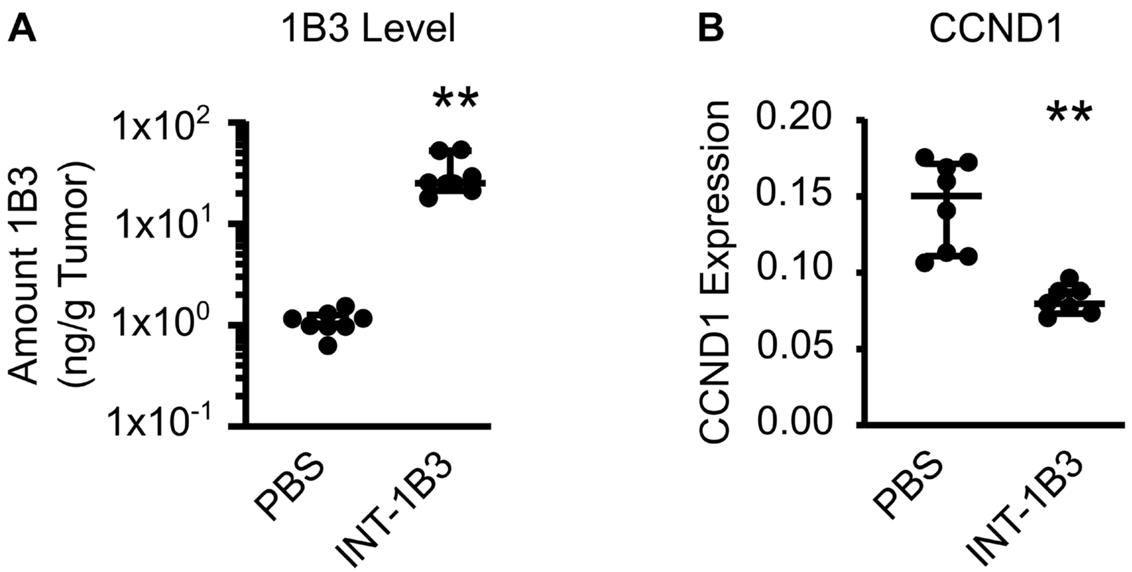

Supplementary Figure 13: Drug delivery and target engagement upon treatment with INT-1B3 in orthotopic human HCC Hep3B tumors. Human HCC Hep3B tumor cells were injected in the flank of Balb/c mice to form subcutaneous tumors. Once appropriate tumor size was reached, tumors were collected, and ~2-mm^3 fragments were implanted into the left liver lobe (orthotopic model) of SCID/Beige mice. Serum AFP levels were measured as indicator of liver tumor development. On day 21, animals were randomized in experimental groups based on increasing serum AFP levels (median ~5.0 µg/mL), and treatment with test items started one day after (*i.e.*, on Day 22). Animals were treated with either vehicle (PBS, i.p.) or INT-1B3 (10 mg/kg/administration, i.v.) daily for three days during the first week, then twice weekly for the following two weeks. Animals were sacrificed on day 49 and tumors snap frozen. RNA was extracted from tumor tissue using TRIzol. (**A**) Stem-loop RT-qPCR was used to detect miR-193a-3p/1B3 levels in treated and PBS groups and the level of expression per ng tumor tissue calculated. The guide (antisense) strand sequence of 1B3 is identical to the mature human miR-193a-3p and the primers therefore cannot distinguish between endogenous miR-193a-3p expression and the level of exogenous 1B3. The y-axis is labelled '1B3 level' but it should be assumed that this also includes endogenous miR-193a-3p expression (which is determined in the PBS-treated conditions). ((**B**) RT-qPCR was used to detect cyclin D1 (*CCND1*) mRNA level, and expression calculated relative to the geometric mean of two reference genes (*UBC* and *RAB7A*) using the $2^{-\Delta CT}$ method. Each point represents an individual animal and error bars show the median with inter quartile range. ** indicates p-value < 0.01 by 2-tailed Mann Whitney test.

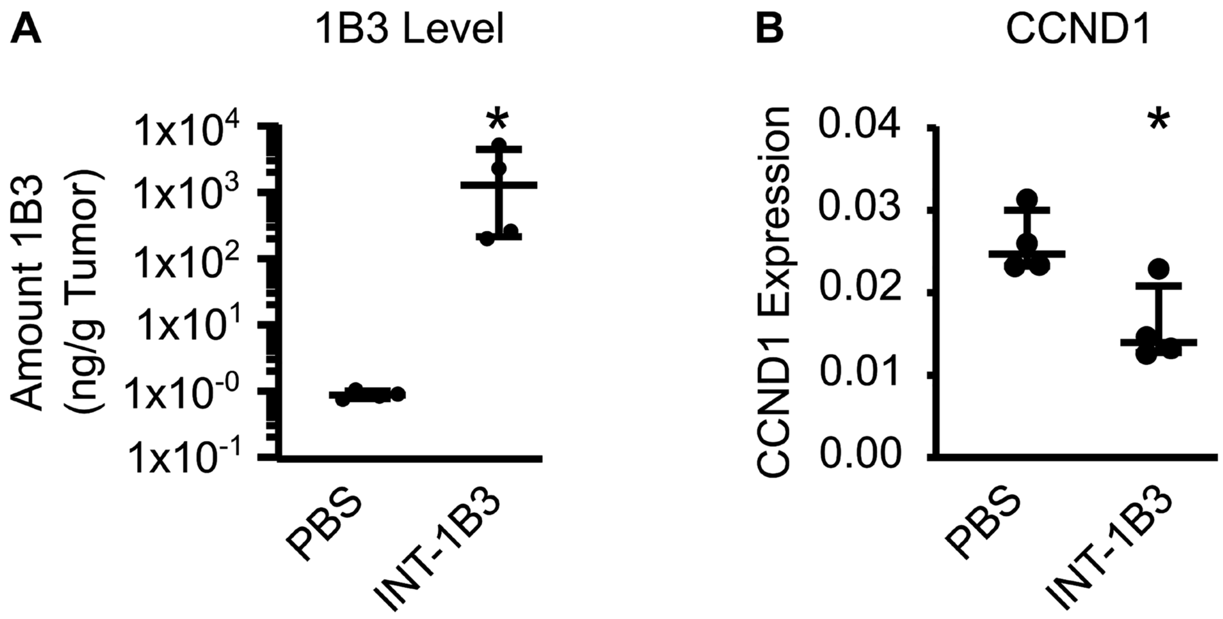

Supplementary Figure 14: Drug delivery and target engagement upon INT-1B3 treatment in subcutaneous human melanoma A2058 tumors. Human melanoma A2058 tumor cells (5×10^6) in 0.1-mL PBS were injected subcutaneously in the flank BALBc/nude mice. After eight days, animals were randomized to experimental groups based on subcutaneous tumor size (median ~150 mm^3; established subcutaneous tumor model). Animals were then treated with either vehicle (PBS, i.p.) or INT-1B3 (3 mg/kg/administration, i.v.) daily for five days the first week, then twice weekly for the next two weeks. Four animals per group were sacrificed 48 h after the fifth injection (*i.e.*, day 7) and tumors snap frozen. RNA was extracted from tumor tissues using TRIzol. (**A**) Stem-loop RT-qPCR was used to detect miR-193a-3p/1B3 levels in INT-1B3- and PBS-treated groups, and the level of expression per ng tumor tissue calculated. The guide (antisense) strand sequence of 1B3 is identical to the mature human miR-193a-3p and the primers therefore cannot distinguish between endogenous miR-193a-3p expression and the level of exogenous 1B3. The y-axis is labelled '1B3 level' but it should be assumed that this also includes endogenous miR-193a-3p expression (which is determined in the PBS treated conditions). (**B**) RT-qPCR was used to detect cyclin D1 (*CCND1*) mRNA level, and expression calculated relative to the geometric mean of two reference genes (*UBC* and *RAB7A*) using the $2^{-\Delta CT}$ method. Each point represents an individual animal and error bars show the median with inter quartile range. ** indicates p-value < 0.01 by 2-tailed Mann Whitney test.

Supplementary Table 1: Cell cycle arrest induced by siRNA

	1B3	siCCND1	siCDK6	siKRAS	siMCL1	siSTMN1	siYWHAZ
H1975	G2/M	No arrest	-	No arrest	-	-	-
Hep3B	G2/M	No arrest	No arrest	No arrest	No arrest	G2/M	No arrest
SNU-449	G2/M	G0/G1	No arrest	No arrest	No arrest	G2/M	No arrest
A2058	G2/M	No arrest	No arrest	No arrest	-	G2/M	No arrest
H1299	G0/G1	G0/G1	G0/G1	No arrest	G0/G1	No arrest	G0/G1

'-' indicates that the siRNA was not tested in that cell line.

Supplementary Table 2: Stem-loop RT-qPCR primers

Primer	Sequence
1B3 Stemloop	GTCGTATCCAGTGCAGGGTCCGAGGTATTCGCACTGGATACGACACTGGGA
1B3 qPCR Forward	TGCCCGAACTGGCCTACAAAGT
1B3 qPCR Reverse	GTGCAGGGTCCGAGGT
U6 Stemloop	GTCATCCTTGCGCAGG
U6 qPCR Forward	CGCTTCGGCAGCACATATAC
U6 qPCR Reverse	AGGGGCCATGCTAATCTTCT

Supplementary Table 3: RT-qPCR primers

Target	Forward Primer	Reverse Primer
B2M	TGTGCTCGCGCTACTCTCT	TGACTTTCCATTCTCTGCTGGA
UBC	CAGCCGGGATTTGGGTCG	CACGAAGATCTGCATTGTCA GT
CCND1	TGCAGCACCTGGCTAAGAAT	TAGCATGCTTCGATGTGGCA
MCL1	TATCTCTCGGTACCTTCGGGA	TTCGTTTTTGATGTCCAGTTTCCG
CDK6	TCCCAGGCAGGCTTTTCATT	TGGCTGGGTTAAATGTCAAACAC
KRAS	TGTGGACGAATATGATCCAACAAT	CCTCTTGACCTGCTGTGTCG
STMN1	CCAGAATTCCCCCTTTCCCC	CCAGCTGCTTCAAGACCTCA
YWHAZ	TGCTTCACAAGCAGAGAGCAA	GGTATGCTTGTTGTGACTGATCG
RAB7A	GTGATGGTGGATGACAGGCTA	GCAGTCTGCACCTCTGTAGAA
